# Dynamic feedback regulation for efficient membrane protein production using a small RNA-based genetic circuit in *Escherichia coli*

**DOI:** 10.1186/s12934-022-01983-2

**Published:** 2022-12-15

**Authors:** Chiara Guidi, Lien De Wannemaeker, Jasmine De Baets, Wouter Demeester, Jo Maertens, Brecht De Paepe, Marjan De Mey

**Affiliations:** Centre for Synthetic Biology, Ghent University, 9000 Ghent, Belgium

**Keywords:** Membrane proteins, *Escherichia coli*, sRNA-based genetic circuitry, Industrial biotechnology

## Abstract

**Background:**

Membrane proteins (MPs) are an important class of molecules with a wide array of cellular functions and are part of many metabolic pathways. Despite their great potential—as therapeutic drug targets or in microbial cell factory optimization—many challenges remain for efficient and functional expression in a host such as *Escherichia coli*.

**Results:**

A dynamically regulated small RNA-based circuit was developed to counter membrane stress caused by overexpression of different MPs. The best performing small RNAs were able to enhance the maximum specific growth rate with 123%. On culture level, the total MP production was increased two-to three-fold compared to a system without dynamic control. This strategy not only improved cell growth and production of the studied MPs, it also suggested the potential use for countering metabolic burden in general.

**Conclusions:**

A dynamically regulated feedback circuit was developed that can sense metabolic stress caused by, in casu, the overexpression of an MP and responds to it by balancing the metabolic state of the cell and more specifically by downregulating the expression of the MP of interest. This negative feedback mechanism was established by implementing and optimizing simple-to-use genetic control elements based on post-transcriptional regulation: small non-coding RNAs. In addition to membrane-related stress when the MP accumulated in the cytoplasm as aggregates, the sRNA-based feedback control system was still effective for improving cell growth but resulted in a decreased total protein production. This result suggests promiscuity of the MP sensor for more than solely membrane stress.

**Supplementary Information:**

The online version contains supplementary material available at 10.1186/s12934-022-01983-2.

## Background

Industrial biotechnology has become key in mitigating today’s main economic and environmental issues through the sustainable and efficient production of enzymes, pharmaceuticals, chemicals, fuels and foods [1, 2]. To this end, employing microorganisms as microbial cell factories (MCFs) shows great promise. To develop an industrial biotechnology process that is scalable and economically viable, extensive host optimization is crucial. These hosts have naturally evolved to be in complete balance with their variable environment which translates into a vast collection of genes, pathways and complex regulatory mechanisms to tightly control both gene expression and enzyme activity [3, 4]. For this reason, maintenance and expression of heterologous pathways in these wild-type (WT) organisms results in low yields or productivities due to the unnatural load on the host cells, e.g., consumption of cellular resources or metabolites or production of toxic intermediates [5–7]. Through metabolic engineering and synthetic biology, both complex native and heterologous biosynthetic pathways can be altered, combined and even expanded to create MCFs which are efficient, productive and robust [8–11].

Despite these achievements, synthetic biology tools are still lagging behind for the efficient expression of membrane proteins (MPs). MPs take up 50% of the cell envelope, where they fulfil central enzymatic reactions, many of which entailing interesting industrial applications. First, MPs play key roles for the design of therapeutic drugs, e.g., membrane-integrated β-glycosyltransferases are implicated in the transfection mechanism of several pathogens. High levels of MP overexpression are required for structural studies and hence development of novel therapeutic targets. More specifically, MPs are involved in several diseases, e.g., cystic fibrosis, cancer and Alzheimer’s disease. Concerning the COVID-19 pandemic and from an engineering perspective, efficient production of biosensing MPs could be a great asset for nanotechnology-enabled solutions to fight COVID-19 and maybe future pandemics [12]. However, MP expression and optimization is also becoming increasingly important for MCF optimization, e.g., to influence substrate uptake or product transport [13, 14]. Secondly, MPs are crucial pathway enzymes for bacterial fermentation of some interesting pharmaceutical or cosmetic products, e.g., hyaluronic acid [15] or flavonoids [16], for which functional MP expression often remains a daunting task.

MPs greatly rely on the cellular membranes for correct folding, structural integrity and activity [17, 18]. Elevated levels of MPs can cause membrane disruption or aggregation in inclusion bodies which makes the isolation of sufficient quantities complicated. Next, heterologous MP expression results in a heavy load on the membrane transport machinery and hence problems with incorporation in the membrane. In addition, titration of protein synthesis precursors to the expression of heterologous MPs lowers MCF viability. In general, MP production often results in only a small amount of well-folded and functional protein, toxicity to the host and little membrane-incorporated protein per cell [17, 18]. One of the most commonly used expression systems for the production of MPs remains *Escherichia coli* (*E. coli*) due to wide knowledge in physiology, fast growth, effective genetic tools and inexpensive culture costs. Several systems that can contribute to resolving part of these MP expression problems are available, e.g., the *E. coli* BL21 (DE3) strain and derivatives together with T7 promoter-based plasmids leading to an optimal level of mRNA of the target gene [19–21], use of a truncated RNase E to stabilise toxicity caused by MP expression [22] or the *E. coli* K12-derived SuptoxD or SuptoxR strains [23] which co-express potent effectors for suppressing membrane protein-induced toxicity. However, these systems deliver only a static solution for optimizing MP expression. Current strains do not sense the stress caused by MP expression. As a result, overall membrane protein expression is lowered without tackling the problem: MP stress.

**Fig. 1:**
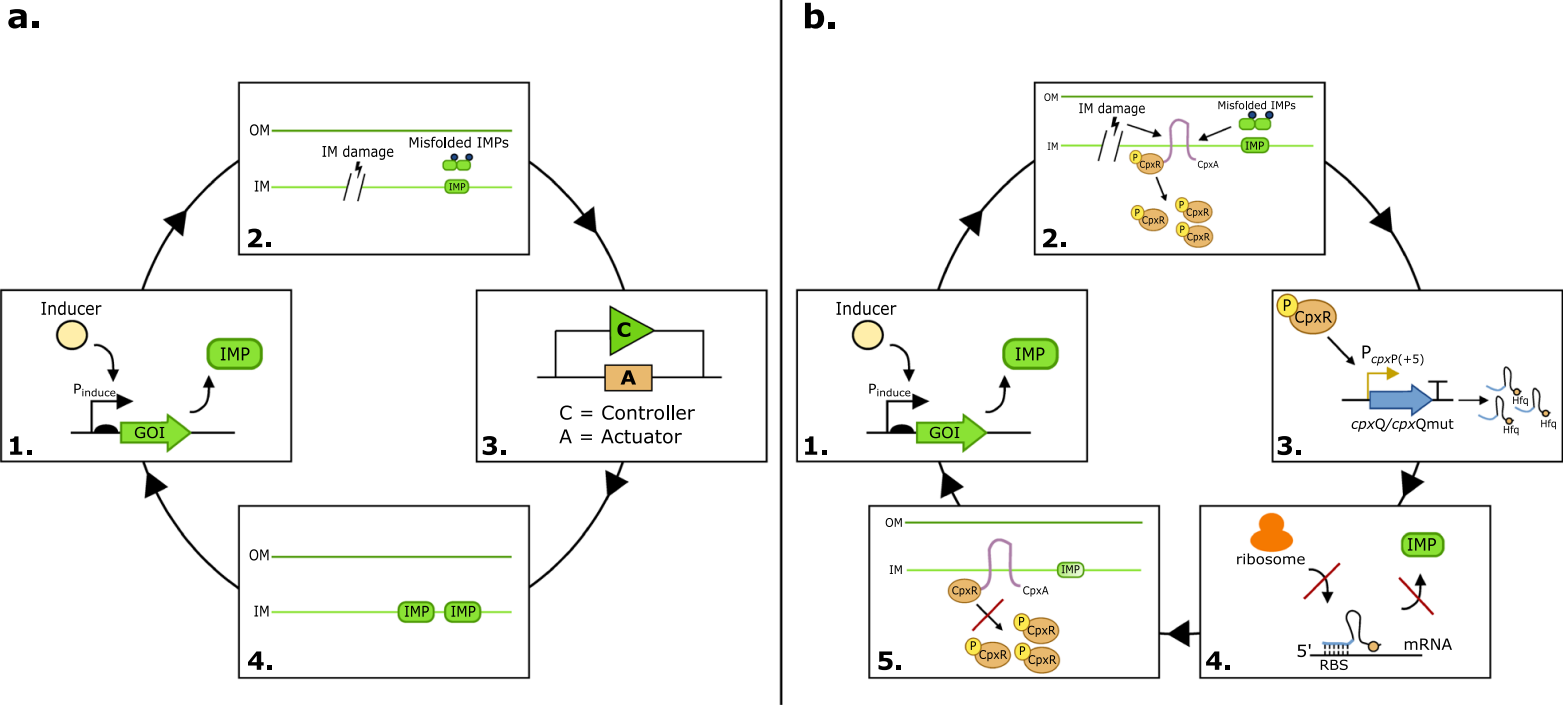
**a** General dynamic regulation of membrane stress. 1. The cells produce an IMP of interest upon addition of the inducer. 2. Accumulation of IMPs causes IM stress signals. 3. A synthetic genetic circuit consisting of a controller and actuator senses and acts upon the IM stress. 4. IM stress diminishes which allows further IMP production. **b** Dynamic regulation of IM stress using a sRNA-based genetic circuit. 1. The cells produce an IMP of interest upon addition of the inducer. 2. Accumulation of IMPs activates the Cpx pathway. 3. The phosphorylated CpxR protein induces the circuit which results in the production of the designed sRNA (*cpx*Qmut). 4. The sRNA represses translation of the IMP. 5. IM stress diminishes and the regulatory systems are downregulated. The amount of sRNA in the cell decreases which allows further IMP production. The cells can now re-enter this cycle. *IM* inner-membrane, *IMP* inner-membrane protein, *OM* outer-membrane, *GOI* gene of interest, *RBS* ribosome binding site

In contrast, dynamic solutions, using genetic circuits, allow for a more efficient, robust and controlled strategy with minimal side-effects on the native metabolism. Genetic circuits enable cells to dynamically respond and perform logic functions upon a certain input signal by influencing the expression of one or multiple enzyme(s) on a transcriptional, translational or post-translational level (Fig. 1a). Although many accomplishments have been established using protein-based, transcriptional regulation [24–27], an attractive alternative are small RNA-based (sRNA) genetic circuits. sRNAs are non-coding RNA-molecules of 50 to 500 nucleotides, highly structured and play a role in the regulatory network of the cell [28]. Most of the sRNAs are directly transcribed from DNA (trans-acting). Others are localized in the 3’ untranslated region (UTR) and are obtained by mRNA processing (cis-acting). By base-pairing with their targets, sRNAs can repress or stimulate translation and transcription elongation and control the stability of transcripts. The use of sRNAs offers many advantages. As sRNAs are not translated into a protein, this results in a faster production, less consumption of cellular resources and lower energy requirement for production. The fast production in combination with a fast degradation rate allows efficient signal propagation, causing an almost linear response curve which is useful for tuning feedback circuits [29, 30]. Additionally, RNA regulators are highly composable, meaning that multiple regulators can be readily combined. Moreover, RNA regulators have relatively simple structures which can, in many cases, be predicted and designed by computational tools [30–32].

To develop a dynamic biological circuit which is fast, composable and enhances the yield of well-folded and functional MP expression in *E. coli*, we sourced and exploited natural regulatory networks and more specifically the envelope stress response regulation mechanisms in *E. coli* for biological DNA parts which can detect membrane stress (i.e., sensor) and respond immediately (i.e., actuator). Several native *E. coli* regulatory systems exist to prevent mistargeting of MPs and associated cell lysis [18, 33–35]. The CpxAR regulatory pathway, further referred to as the Cpx pathway, is a two-component system in *E. coli* that senses and acts on a variety of signals coming from the inner membrane (IM) [36–40]. This compelling system was therefore exploited to create a control loop to dynamically control MP expression in view of increasing the yield of well-folded and functional MPs (Fig. 1b). Therefore, the DNA parts of this CpxAR regulatory system (i.e., promoter and sRNA) were evaluated and adapted to build a dynamically regulated negative feedback mechanism. The usefulness was evaluated for the expression of different MPs in *E. coli*.

## Results

### A membrane stress sensor: evaluation of promotor P_***cpx***P_

**Fig. 2:**
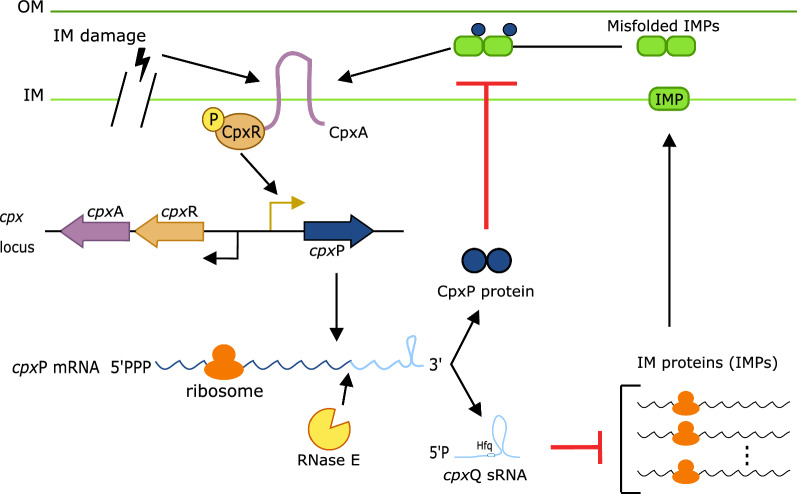
Native regulatory CpxAR two-component system countering IM stress in *Escherichia coli* (*E. coli*). Triggered by misfolded IMPs and IM damage, structural changes in the CpxA sensor domain cause the histidine kinase domain located in the cytoplasm to autophosphorylate. CpxA subsequently phosphorylates CpxR, the response regulator, activating it for transcriptional regulation. One of the activated genes encodes CpxP, which inhibits CpxA activation and functions in a negative-feedback loop. In parallel, the *cpx*P mRNA is further processed by RNase E to form the small RNA *cpx*Q that in turn post-transcriptionally reduces the synthesis of IMPs in the cytoplasm. *IM* inner membrane, *OM* outer membrane, *IMP* inner membrane proteins

The Cpx system consists of the membrane-anchored sensor kinase CpxA, the response regulator CpxR and the periplasmic protein CpxP (Fig. 2). Under IM stress conditions, CpxR is phosphorylated by CpxA and activates the transcription of several genes including the *cpx*P gene. The transcribed *cpx*P mRNA is translated to make the CpxP protein and is further processed by RNase E at the 3’UTR to produce *cpx*Q sRNA. Whereas CpxP acts in the membrane to degrade misfolded IM proteins (IMPs) and regulate CpxA activity at the post-translational level, *cpx*Q together with the Hfq protein post-transcriptionally reduce the synthesis of IMPs in the cytoplasm [36].

**Fig. 3:**
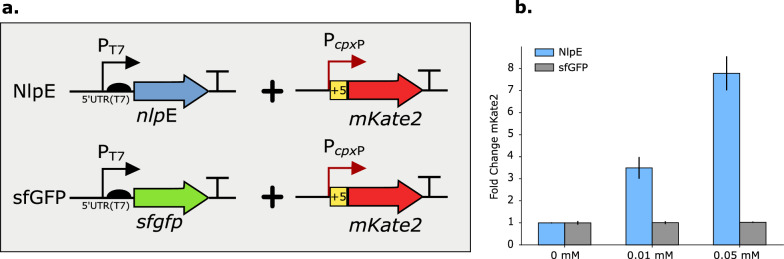
**a** Principle of the indirect stress sensor using positive and negative control to trigger IM stress. **b** Relative mKate2 fluorescence of an equal number (OD_600_ = 0.3, mid-exponential phase) of *Escherichia coli* (*E. coli*) DE3 cells (See Additional file 1: Table S2 for more details) producing either NlpE (IMP of *E. coli*, positive control) or sfGFP (cytoplasmatic superfolder green fluorescent protein, negative control) for several IPTG-concentrations. The fluorescence of NlpE and sfGFP producing DE3 cells at 0 mM IPTG is arbitrarily set to one and fluorescence of other IPTG-concentrations was compared to this value. IM = inner-membrane, IMP = inner-membrane protein, mKate2 = red fluorescent protein

The ability to sense membrane stress could provide a first insight into the potential damage caused by interference with the membrane integrity upon (heterologous) MP expression. Furthermore, membrane stress would be an ideal input trigger to dynamically regulate MP expression. Upon IM stress, phosphorylated CpxR (CpxR-P) binds to DNA to regulate transcription of about 70 genes [37, 41]. The activity of the *cpx*P promoter (P_*cpx*P_) was chosen and evaluated as a biosensor for IM-related stress using the red fluorescent reporter protein mKate2 [42] (Fig. 3a). The P_*cpx*P_ promoter is recognized by the sigma 70 factor, generally known as the major or primary sigma factor, which is essential for general transcription in exponentially growing cells. In addition, three CpxR-P binding sites were identified of which one is located up to five nucleotides directly downstream of the transcription start site (TSS) [43]. Therefore, the promoter—including these five nucleotides—used for this indirect analysis and further experiments will be referred to as P_*cpx*P(+5)_.

Overexpression of the lipoprotein NlpE served as positive control as it is known membrane protein and proven trigger of the Cpx pathway [36, 39, 40, 43, 44]. As negative control, expression of the cytoplasmic fluorescent protein sfGFP [45] was chosen. Both proteins were controlled by the T7 expression system, in order to induce protein synthesis at a specific moment in time. Expression of NlpE lead to a sevenfold increase in mKate2 fluorescence for an IPTG-concentration of 0.05 mM which indicated the ability of the P_*cpx*P(+5)_ promoter to detect membrane stress (Fig. 3b). In contrast, the negative control, cytoplasmic sfGFP expression, did not result in a significant mKate2 increase (p = 0.316, Fig. 3b and Additional file 1: Table S1 and Fig. S8). In addition, the ideal IPTG-concentration was found to be ranging from 0 to 0.2 mM due to the high levels of metabolic burden and cell lysis above 0.2 mM (Additional file 1: Fig. S1).

### The actuator: cpxQ sRNA engineering

As shown in Fig. 2, the Cpx pathway consists of a trans-encoded sRNA, *cpx*Q, which recognizes designated binding sites in target mRNAs of proteins with predominant periplasmic or IM localization [46, 47]. The region of complementarity between the sRNA and the mRNA of the protein, i.e., the seed region, determines the efficiency of silencing. In *E. coli*, one of these target mRNAs is *nha*B, which translates into a sodium-proton antiporter, for which the *cpx*Q seed region is fully known [36]. This seed region can bind both to part of the 5’UTR and coding sequence of *nha*B (See Additional file 1: Fig. S2 for *cpx*Q seed regions to *nha*B mRNA).

**Fig. 4:**
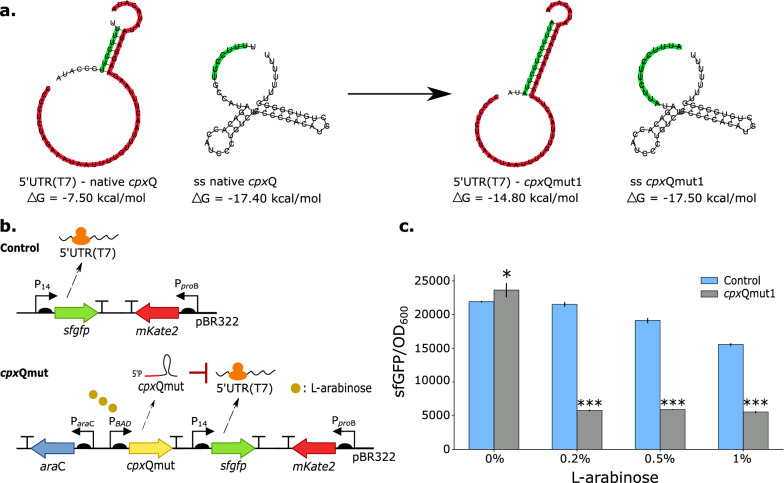
**a** Interaction between part of the native and rationally designed *cpx*Qmut1 (marked green) and the 5’UTR(T7) (marked red). Gibbs free energy (ΔG) of the interaction is depicted. The secondary RNA structure of both the native and rationally designed *cpx*Qmut1 with ΔG-value are also illustrated. **b** Designs of control plasmid without engineered *cpx*Q sequence and plasmid with *cpx*Qmut1 sequence controlled by the inducible promoter P_BAD_. **c** Bar plots representing the output sfGFP/OD_600_ for the control plasmid (blue) and the plasmid expressing the sRNA *cpx*Qmut1 (grey) and this for different L-arabinose concentrations. sfGFP/mKate2 values for an equal number (OD_600_ = 0.4, mid-exponential phase) of *Escherichia coli* DH10B cells are plotted. All experiments were carried out in replica triplicates (biological variation) and the error bars represent one standard deviation from the mean value. * = p-value < 0.05, *** = p-value < 0.001 obtained by conducting a two-sample t-test between strains expressing the control plasmid and strain expressing the sRNA *cpx*Qmut1 and this for each L-arabinose concentration, *araC* regulator of the P_BAD_ promoter, *mKate2* red fluorescent protein, *OD*_*600*_ optical density measured at 600 nm, *pBR322* medium-copy origin of replication (~ 15–20 copies per cell), *RBS* ribosome binding site, *sfGFP* superfolder green fluorescent protein, *5’UTR(T7)* 5’ untranslated region of T7 expression system, sRNA = small RNA, *TIR* translation initiation region, *ss* secondary structure

As sRNAs are known to facilitate rapid, reversible, dynamic and efficient signal propagation, the ability to alter these seed regions to bind new and specific targets could be a powerful tool for the purpose of dynamic pathway regulation. In a first step, the known seed region of the native *cpx*Q sequence (Fig. 4a) was designed to specifically recognize a 5’UTR of interest, i.e., the 5’UTR(T7), which is typically used in protein expression plasmids [48, 49]. The better the complementarity, the better the sRNA binds to this translation initiation region (TIR) and the more stable the sRNA-mRNA complex is, which is reflected by the Gibbs free energy of the sRNA-mRNA complex (ΔG_1_). Next to the sRNA-mRNA complex, the sRNA stability (reflected by the Gibbs free energy of the secondary structure of the unbound sRNA (ΔG_2_)) is an important parameter to avoid rapid degradation and successful targeting of the mRNA as the sRNA has to remain able to interact with the RNA-binding protein Hfq [50–56]. The RNA secondary structure and interaction with 5’UTR(T7) were constructed using RNAcofold [57]. By adding mismatches to the seed region of the *cpx*Q sRNA (Additional file 1: Table S3), the ΔG_1_ of the sRNA-mRNA complex was varied to in silico create an engineered *cpx*Q sRNA (*cpx*Qmut1) with improved binding to the 5’UTR(T7) (Fig. 4a). In addition, the secondary structure of *cpx*Qmut1 closely resembles the native *cpx*Q structure to ensure sRNA stability due to optimal interaction with Hfq.

As proof of concept that the engineered sRNA *cpx*Qmut1 can specifically in vivo recognize the 5’UTR(T7), regulating the expression of the fluorescent protein sfGFP [45], a plasmid was introduced in *E. coli* containing a sfGFP under control of the constitutive promoter P_14_ [58] and 5’UTR(T7) and the designed *cpx*Qmut1 controlled by the P_BAD_ promoter [59] (Fig. 4b). Constitutive expression of the red fluorescent protein mKate2 [42] was used as a reference to account for extrinsic factors influencing gene expression (such as cell growth). A plasmid without the designed sRNA sequence served as a control (Fig. 4b). It is important to note that for both strains—control and *cpx*Qmut1—the native *cpx*Q sRNA is still present as being part of the native CpxAR-two component system in *E. coli*. No knockout strains were considered in this set-up. Fluorescence and OD_600_ were measured over time for different L-arabinose concentrations. A significant decrease in fluorescence per cell was observed indicating the ability of the engineered sRNA *cpx*Qmut1 to specifically bind to 5’UTR(T7) and prohibit further translation (Fig. 4c and Additional file 1: Fig. S1 and Table S4).

### Evaluation of a dynamically regulated sRNA-based genetic circuit

**Fig. 5:**
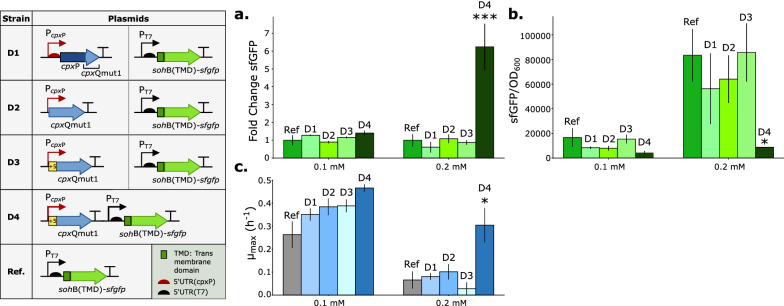
Comparison of four different sRNA-based (inner-membrane) stress circuit designs on growth and protein production, in relation to the original SohB(TMD)-sfGFP expressing *Escherichia coli* cells as a reference. **a** Total sfGFP results for overexpression of membrane targeted sfGFP (inducer concentrations of 0.1 and 0.2 mM IPTG). The fluorescence of SohB(TMD)-sfGFP producing *Escherichia coli* (*E. coli*) MG1656 DE3 cells (Additional file 1: Table S2) without stress circuit was arbitrarily set to one and fluorescence of the same strains with respectively D1-4 was compared to this value (Timepoint: 10 h, early stationary phase). **b** Bar plots representing the output sfGFP/OD_600_ for overexpression of membrane targeted sfGFP (inducer concentrations of 0.1 and 0.2 mM IPTG). sfGFP/OD_600_ values for an equal number (OD_600_ = 0.2) of *E. coli* MG1656 DE3 cells were plotted. **c** Specific growth rate (µ_max_) for strains (*E. coli* MG1656 DE3) expressing Ref and D1-4, respectively (inducer concentrations of 0.1 and 0.2 mM IPTG). Error bars represent standard deviations based on 3 biological replications. For statistical comparison, a one-way ANOVA was performed. * = p-value < 0.05, *** = p-value < 0.001, *IPTG* isopropyl β-D-1-thiogalactopyranoside, *TMD* transmembrane domain, *UTR* untranslated region, *SohB* inner-membrane protein from *E. coli,* *sfGFP* superfolder green fluorescent protein

The promoter P_*cpx*P(+5)_ and the rationally designed sRNA *cpx*Qmut1 were combined in one genetic circuit to achieve membrane stress-driven dynamic regulation: sensing stress resulting from MP overexpression, and subsequently regulating the synthesis of these proteins to reduce their toxicity. To this end, four different designs were constructed and compared in order to aid the cells combat membrane-related stress signals and hence increase MP production (Fig. 5). The four designs were evaluated for the efficient production of a membrane-targeted sfGFP [45]. The use of fluorescence as a quantification of membrane integration has been proven in the past, even for analysis of highly complex transmembrane (TM) proteins [60–62]. Here, the fluorescent protein sfGFP was targeted to the membrane using the *E. coli* transmembrane domain (TMD) of the IM protein SohB [63, 64]. The SohB TMD was selected from a list of different bacterial and human TMDs and signal peptides which were tested for their capacity to guide sfGFP to the *E. coli* membrane (Additional file 1: Fig. S4). Of note is that both the signal peptide of PelB, commonly used to target proteins to the periplasm of *E. coli* [49], and the TMD of 17aCYP, commonly used to target proteins like cytochrome P450 enzymes to the IM of *E. coli* [16], yield cytoplasmatic sfGFP instead of cell membrane targeted sfGFP.

For the first three designs (D1-D3), a dual plasmid system was created comprising a low copy plasmid (pSC101 *ori*), bearing the *cpx*Qmut1 sRNA expression operon, and a medium copy plasmid (pBR322 *ori*), expressing SohB(TMD)-sfGFP under control of the promoter P_T7_ and the 5’UTR(T7) [48, 49], on which the *cpx*Qmut1 sRNA can bind, hindering translation. Design D1 is based on the natural processing of the *cpx*P mRNA in the CpxAR two-component system in *E. coli*: upon activation of the stress-sensitive promoter P_*cpxP*_, *cpx*P mRNA is transcribed and further processed by the RNase E to produce *cpx*Q sRNA [36, 52]. For this, in design D1 the 3’ end of the native *cpx*P mRNA was altered to match the sequence of *cpx*Qmut1. Design D2 circumvents RNase E cleavage and immediately transcribes *cpx*Qmut1 sRNA upon promoter P_*cpx*P_ activation. Finally, in design D3 the first five nucleotides of the *cpx*P mRNA were fused with the *cpx*Qmut1 sRNA. The preservation of these first five nucleotides after the transcription start site in the *cpx*P operon was found to be important for CpxR-P binding to the third CpxR-P binding site in the P_*cpx*P_ promoter [43]. All dual plasmid constructs were introduced into *E. coli* MG1656 DE3 cells (Additional file 1: Table S2). The effect of the three designs (D1-D3) on cell growth and fluorescence was compared to the reference strain that expresses SohB(TMD)-sfGFP in absence of *cpx*Qmut1 sRNA expression (Ref), for two different IPTG-concentrations (Fig. 5a–c). Although no significant differences were noted for maximum specific growth rate, total protein production or protein production per cell, an average increase in final OD_600_ was observed for an inducer concentration of 0.1 mM (Additional file 1: Fig. S5a and Table S5).

To further optimize this sRNA-based membrane stress circuit design, we investigated the response to an important genetic parameter for sRNAs: the impact of the sRNA-mRNA ratio [65, 66], as an excess of sRNA is often required to saturate binding of the target mRNA. Additionally, tight spatial correlation of the sRNA and its target could be important to increase effective binding due to a decrease in search space of the sRNA for its target [29, 67]. This local concentration burst of sRNA will likely avoid the short-circuiting of other Hfq-mediated regulatory networks as the Hfq chaperone is a limiting factor in the bacterial cell [51]. For this reason, design D3 of the sRNA-based (IM) stress circuit was transferred to the reference plasmid (SohB(TMD)-sfGFP). Application of the stress circuit by the single plasmid design (D4) significantly reduced protein production per cell, indicating the functionality of the circuit (Fig. 5b). Additionally, there was a significant improvement in cell growth and a sixfold increase in total protein production (one-way ANOVA, p = 0.018 and p = 0.001, respectively) in contrast to the double plasmid designs (D1-3) (Fig. 5a, c).

### Smart sRNA library for increased membrane protein production

To rationally design a sRNA to specifically bind a target, two important parameters need to be considered: sRNA stability and sRNA-mRNA interaction. Therefore, a library of nine different variants of the native *cpx*Q was created to find the optimal *cpx*Q with the best balance between a stable secondary structure (ΔG_2_) and interaction with the 5’UTR(T7) (ΔG_1_). To this end, the library is called ‘smart’ as several combinations of both parameters were made, e.g., strong sRNA-mRNA interaction but low sRNA stability or stable sRNA secondary structure but intermediate sRNA-mRNA interaction (Table 1). In this way, the smart library covers a relevant range of both parameters. The sRNAs *cpx*Qmut1, *cpx*Qmut2 and *cpx*Qmut3 were designed to have a secondary structure which closely resembled to the native *cpx*Q but with higher complementarity to the 5’UTR(T7). Next, for *cpx*Qmut4 the region of complementarity increased, improving the ΔG_1_ value, although ΔG_2_ was close to the native *cpx*Q but with different folding characteristics. Finally, sRNAs *cpx*Qmut5-9 have an expanded region of complementarity to 5’UTR(T7), resulting in decreased ΔG_1_s, up to − 40.07 kcal/mol [68]. However, for *cpx*Qmut7-8 this resulted in a more unstable secondary structure (Additional file 1: Fig. S6). This smart sRNA library was evaluated for the efficient production of a membrane-targeted sfGFP (SohB(TMD)-sfGFP). The vectors expressing the native *cpx*Q or the respective *cpx*Qmut variant are similar to design D4 (Fig. 5, one plasmid design) for which *cpx*Qmut1 was replaced by one of the other nine *cpx*Q(mut) sequences.

**Table 1:** Gibbs free energies (ΔG) of the sRNA-mRNA complex and secondary structures of the unbound sRNA

	ΔG_1_ (kcal/mol) sRNA-mRNA	ΔG_2_ (kcal/mol) Secondary structure
Native *cpx*Q	− 4.29	− 17.40
*cpx*Qmut1	− 11.36	− 17.50
*cpx*Qmut2	− 12.04	− 16.50
*cpx*Qmut3	− 12.12	− 18.50
*cpx*Qmut4	− 23.46	− 14.40
*cpx*Qmut5	− 26.94	− 14.40
*cpx*Qmut6	− 30.20	− 14.80
*cpx*Qmut7	− 39.52	− 6.40
*cpx*Qmut8	− 40.07	− 6.20
*cpx*Qmut9	− 33.90	− 14.40

ΔG_1_ (kcal/mol) gives the stability of the sRNA-mRNA complex. ΔG_2_ (kcal/mol) gives the ΔG of the secondary structure of the free *cpx*Q sRNA

*mRNA* messenger RNA, *sRNA* small RNA

**Fig. 6:**
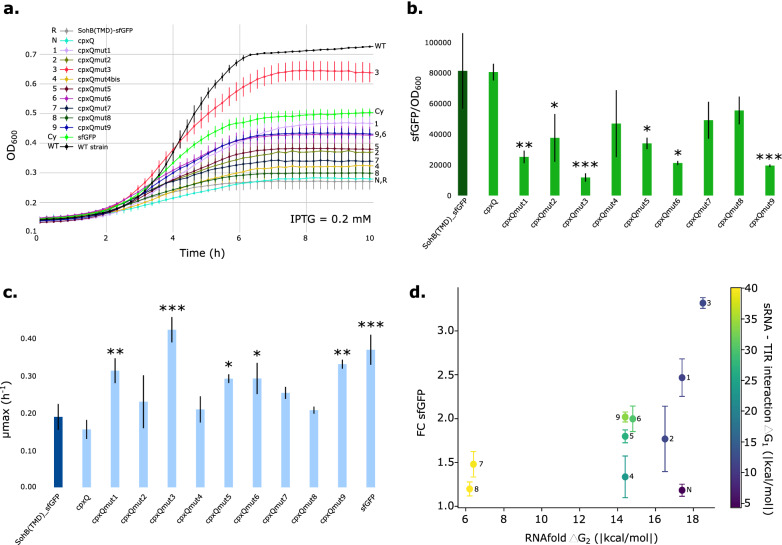
Effect of the smart library of sRNA-based stress circuits on cell growth and protein production of a membrane targeted sfGFP (SohB(TMD)-sfGFP). The strain which expressed construct SohB(TMD)-sfGFP without stress circuit, was used as reference (R). The strain with native *cpx*Q served as negative control (N). Expression of sfGFP in the cytoplasm was also included (Cy). **a** OD_600_ measured in time. **b** sfGFP/OD_600_-values of the different strains (inducer concentration 0.2 mM IPTG). sfGFP/OD_600_ values for an equal number (OD_600_ = 0.25) of *E. coli* MG1656 DE3 cells were plotted. This ratio was corrected for background fluorescence and optical density of the medium and the cell culture (*Escherichia coli* MG1656 DE3). **c** Maximum specific growth rate (µ_max_) of the different strains. The experiment was carried out in triplicate (biological variation) and the error bars represent one standard deviation from the mean value. For panel b and c, significant differences between strains expressing construct SohB(TMD)-sfGFP and all constructs with native or mutated *cpx*Q were calculated using one-way ANOVA. **d** Scatter plot representing fold change of sfGFP in function of the ΔG_2_ absolute value for the secondary sRNA structure (Timepoint: 10 h, stationary phase). Complete plasmid details can be found in Additional file 1: Table S6. The colormaps represents the ΔG_1_ absolute value. * = p-value < 0.05, ** = p-value < 0.01, *** = p-value < 0.001, *ΔG*_*1*_ Gibbs free energy for sRNA-mRNA interaction, *ΔG*_*2*_ Gibbs free energy for sRNA stability, *sfGFP* superfolder green fluorescent protein, *SohB(TMD)* transmembrane domain of inner-membrane protein SohB from *E. coli*, *TIR* translation initiation region

Again, fluorescent intensity was used as an indication for the amount of protein targeted to the membrane. A strain producing cytoplasmic sfGFP served as reference for fluorescence production without membrane stress. Both OD_600_, as a measure of cell growth, and green fluorescence, as a measure of protein production, were continuously followed over time (Fig. 6a and Additional file 1: Fig. S7a). Targeting sfGFP to the membrane, without the sRNA-based circuit, significantly reduced cell growth and fitness (Fig. 6a–c and Additional file 1: Table S5). Cytoplasmic sfGFP expression decreased cell growth as well, probably due to metabolic burden issues (µ_max_ of 0.37 ± 0.04 h^−1^ for cytoplasmic sfGFP expression compared to 0.54 ± 0.02 h^−1^ for *E. coli* MG1656 DE3 cells without plasmid). Overall, addition of the stress circuit positively affects cell growth and total protein production (Fig. 6a and Additional file 1: Fig. S7). Overexpression of the native *cpx*Q sRNA, as a result of the applied membrane stress, did not significantly improve cell growth or protein production, highlighting the orthogonality of the other rationally designed sRNAs for 5’UTR(T7). Rationally designed sRNAs *cpx*Qmut1, 3, 5, 6 and 9 resulted in a significant increase in µ_max_ compared to the µ_max_ of the strain without stress circuit (SohB(TMD)-sfGFP). The same could be concluded from sfGFP intensity measurements (Additional file 1: Fig. S7b). Overall, rationally designed sRNAs *cpx*Qmut1 and 3 performed significantly better compared to the other rationally designed *cpx*Q sRNAs (ΔG_2_ of − 17.50 and − 18.50 kcal/mol, respectively). More specifically, the decreased protein production per cell (Fig. 6b) resulted in an improved growth rate and subsequently increased total protein production (Fig. 6d). The balance between sRNA stability and complementarity to 5’UTR(T7) resulted in significantly increased maximum specific growth rates (p = 0.009 and p = 0.001, respectively, Additional file 1: Table S5), total protein production (p = 0.001 and p = 0.001, respectively, Additional file 1: Table S5) and final OD_600_ values (Fig. 6a). Therefore, these two sRNAs were chosen in further evaluation of the stress circuit for the overexpression of MPs.

### Dynamic regulation for the overproduction of membrane proteins

**Fig. 7:**
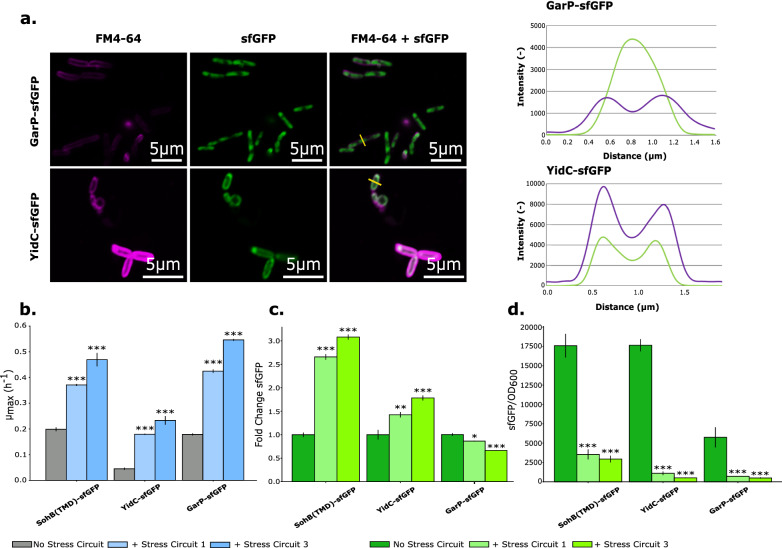
Effect of the sRNA-based stress circuit on protein production of functional MPs expression. **a** Confocal scanning light microscopy images with intensity cross-section profiles for both the red FM4-64 membrane dye (purple colour) and the green fluorescent protein signal (GFP, green colour). Yellow bars represent the position used to make a cross-section profile. **b** Specific growth rate (µ_max_) for strains (*Escherichia coli* (*E. coli*) MG1656 DE3, Additional file 1: Table S2) expressing SohB(TMD)-sfGFP, YidC-sfGFP and GarP-sfGFP. **c** Total protein production results for overexpression of proteins, SohB(TMD)-sfGFP, YidC-sfGFP and GarP-sfGFP, localized in the membrane. The fluorescence of SohB(TMD)-sfGFP, YidC-sfGFP and GarP-sfGFP producing *E. coli* MG1656 DE3 cells without stress circuit was arbitrarily set to one and fluorescence of the same strains with respectively stress circuits 1 and 3 were compared to this value. **d** sfGFP/OD_600_-values for overexpression of proteins, SohB(TMD)-sfGFP, YidC-sfGFP and GarP-sfGFP with and without stress circuit. sfGFP/OD_600_ results for an equal number (OD_600_ = 0.25 for GarP-sfGFP and SohB(TMD)-sfGFP, OD_600_ = 0.18 for YidC-sfGFP) of *E. coli* MG1656 DE3 cells were plotted. This ratio was corrected for background fluorescence and optical density of the medium and the cell culture (*Escherichia coli* MG1656 DE3). All experiments were carried out in triplicates (biological variation) and the error bars represent the standard deviation from the mean value. Statistical difference between strains expressing the MP without and with stress circuit 1 or 3, was calculated using a one-way ANOVA. * = p-value < 0.05, ** = p-value < 0.01, *** = p-value < 0.001, IPTG (0.2 mM) was added to induce protein expression. YidC = membrane protein insertase in *E. coli*, GarP = TM transporter in *E. coli*, MP = membrane protein, sfGFP = superfolder green fluorescent protein, SohB(TMD) = transmembrane domain of inner-membrane protein SohB from *E. coli*, Stress circuit 1 (mut1) = strain expressing MP and sRNA *cpx*Qmut1, Stress circuit 3 (mut3) = strain expressing MP and sRNA *cpx*Qmut3

Next, the optimized stress circuits (design D4 with *cpx*Qmut1/mut3) were applied for the overexpression of functional MPs. For this purpose, two bacterial MPs were chosen: GarP and YidC (Additional file 1: Table S7). GarP is a TM transporter, probably of galactarate and D-glucarate. No crystal structure for this protein is yet obtained but homology modelling predicts 12 TMDs with a cytoplasmic loop between TMD6 and 7. Both the N- and C-termini are located in the cytoplasm [69]. The second MP, YidC, is a MP insertase. This protein is inserted with six TMDs in the *E. coli* IM and contains a large periplasmic loop between TMD1 and 2. YidC plays an important role in the correct folding of MPs, both dependent and independent of the Sec-translocon [70]. Localization of both proteins was checked with the Zeiss LSM 780 confocal scanning light microscope with Airyscan technology. Both proteins were fused to sfGFP to allow visualization. In contrast to what was suggested in literature, GarP overexpression resulted in the aggregation of GarP in the cytoplasm. YidC was predominantly localized in the membrane with some aggregation at the poles (Fig. 7a). In both cases, elongated cells were observed and were able to significantly activate the membrane stress sensor (Additional file 1: Fig. S8 and Table S8). In analogy with *E. coli* overexpressing membrane-targeted sfGFP, overexpression of GarP and YidC severely hampered cell growth and hence total protein production (Additional file 1: Fig. S9). Co-expression of both MPs with the optimized stress circuit (design D4 with *cpx*Qmut1/mut3) was evaluated. Both OD_600_ and green fluorescence intensity were measured in time. The TIR (5’UTR(T7)) of the two MPs was the same as for the membrane-targeted sfGFP.

For the overexpression of all proteins, growth curves regained the sigmoid shape and a higher final OD_600_ was obtained (Additional file 1: Fig. S9). These results were further reflected in the µ_max_-values: application of the stress circuit with sRNAs *cpx*Qmut1 and 3 significantly increased µ_max_ (Fig. 7b and Additional file 1: Table S5) and decreased protein production per cell (Fig. 7d). Additionally, adding the stress circuit to the overexpression of YidC resulted in a significant increase in total production (Fig. 7 c) and Additional file 1: Table S5). Similar to the results obtained with the smart sRNA library (Fig. 6), *cpx*Qmut3 sRNA performed better than *cpx*Qmut1 (Fig. 7b, c). Although, a notable difference was observed depending on the source of stress. Direct GarP visualization (Fig. 7a) indicated cytoplasmic localization in aggregates. Overexpression of the latter protein in combination with the stress circuit, resulted in a significant decrease in protein productivity (Fig. 7c and Additional file 1: Table S5). Finally, the influence of both circuits on protein localization or membrane insertion was visualized with the Airyscan technology. In contrast to the improved cell growth and productivity results, no effect on localization or cell physiology was observed. Cells retained an elongated form and in the case of GarP overexpression, aggregation still occurred (Additional file 1: Fig. S10). To demonstrate the specificity of *cpx*Qmut1 and 3, a non-specific *cpx*Qmut (ΔG_1_ = − 3.94 kcal/mol for binding with the 5’UTR of interest) was designed. The non-specific *cpx*Qmut sRNA (Additional file 1: Fig. S11a) could not significantly increase µ_max_ (Additional file 1: Fig. S11b) and did not significantly decrease protein production per cell (Additional file 1: Fig. S11c).

## Discussion

In this study, a dynamically regulated sRNA-based circuit was developed to counter stress caused by MP production. Due to the many advantages connected to the use of sRNAs in metabolic engineering, we designed and built a dynamic, membrane stress-driven, negative feedback circuit that combined the use of sRNAs with the membrane stress-sensitive promoter P_*cpx*P(+5)_ [29, 68, 71, 72]. The sRNA *cpx*Q was designed to specifically bind to a 5’UTR of interest (5’UTR(T7)) which controlled expression of the fluorescent protein sfGFP. A significant decrease in fluorescence per cell was observed, suggesting the ability of our engineered sRNA to specifically bind to the 5’UTR(T7) and prohibit further translation. Three different sRNA-based circuit designs were assessed for their ability to improve cell growth and/or total membrane-targeted sfGFP production. All three tested dual plasmid designs (D1-D3) had an average beneficial effect on cell growth but not on membrane-targeted sfGFP production for 0.1 mM IPTG induction. At a higher level of induction (0.2 mM IPTG), no positive effect on cell growth or protein production was observed for D1-D3. Multiple hypotheses could clarify these observations. First, D1 needs an additional step to fully activate the circuit: cleavage of *cpx*P mRNA by RNase E to form *cpx*Qmut1 sRNA. This extra step possibly delayed the speed at which the circuit could react to membrane-related stress. It seemed unlikely that RNase E did not recognize the correct cleavage site as this was not altered in the sRNA design process [52]. Additionally, in D1 *cpx*P mRNA was translated to CpxP which could cause an allocation of resources without any added benefit for the cells. Moreover, CpxP is known for its repressing activity of the Cpx pathway and hence decreases the amount of CpxR-P [73–76]. Designs D2 and D3 skip this RNase E cleavage step and directly produce the required sRNA. For D2, an incomplete interaction between CpxR-P and the *cpx*P promoter could have occurred. The extra 5-nucleotide extension between the P_*cpx*P_ promoter and the sRNA sequence in D3 should allow for an optimal CpxR-P—P_*cpx*P_ interaction however no significant improvement in cell growth and total protein production was observed [43, 77, 78]. For this reason, a final important parameter was investigated: the impact of the sRNA-mRNA ratio. Tight spatial correlation of the sRNA and its target could be important to increase effective binding due to a decrease in search space of the sRNA for its target and/or by preventing Hfq short-circuiting [79]. Therefore, a single plasmid design (D4) in contrast to the dual plasmid design (D3) of the sRNA-based (membrane) stress circuit was evaluated (Fig. 5). The single plasmid design—in contrast to the dual plasmid design—of the sRNA-based (membrane) stress circuit successfully improved cell growth and membrane-targeted sfGFP production (Fig. 5a–c).

Next, a smart library of different *cpx*Q sRNAs was designed and evaluated, using D4, for the optimization of membrane-targeted sfGFP production. These *cpx*Q sRNA variants differed in binding strength to the 5’UTR(T7) (region of complementarity) and secondary structure. The application of the stress circuits resulted in (partly) restored growth rate, cell fitness and increased total protein production (Fig. 6 and Additional file 1: Fig. S7). As protein production per cell did decrease, this indicated the ability of the circuit to dynamically regulate overexpression of the membrane-targeted protein sfGFP (Fig. 6b). However, we want to stress the promiscuity and difficulty for interpreting protein production per cell. More specifically, overexpression of the selected membrane proteins resulted in severe growth rate retardation and final OD_600_-values and therefore complicating a completely correct comparison of protein production per cell at a pre-set moment on the growth curve, i.e., exponential or stationary phase. After reaching the stationary phase, productivity stopped improving. The P_*cpx*P_ promoter has been reported to be activated in response to entry into stationary phase and hence, negative feedback is activated and protein production halted [40]. Altogether, similar trends were observed for most of the designed sRNAs: improved cell growth and total protein production. As expected, the non-optimized, native *cpx*Q sRNA exerted no beneficial effect on both growth and total protein production due to the inability to target our 5’UTR(T7). However, increased expression of the native *cpx*Q could still influence other *E. coli* proteins but not to an extent that stress levels were relieved. The best performing *cpx*Qmut sRNAs (*cpx*Qmut1 and 3) had better complementarity to the 5’UTR(T7) and a secondary structure closely resembling the native *cpx*Q structure (Table 1 and Additional file 1: Fig. S6). In both sRNAs the Rho-independent terminator region, required for interaction with Hfq, is unchanged and available for association with the RNA-chaperone Hfq. The sRNAs *cpx*Qmut5, 6 and 9 also significantly increased cell growth and overall protein yield (p = 0.048, p = 0.046 and p = 0.002, respectively, Additional file 1: Table S5). Even though these three sRNAs have better complementarity to the 5’UTR(T7), e.g., ΔG_1_ = − 33.90 kcal/mol for *cpx*Qmut9 compared to ΔG_1_ = − 12.12 kcal/mol for *cpx*Qmut3, the influence on growth and total protein production was less pronounced than for *cpx*Qmut1 and 3 (Fig. 6c and Additional file 1: Fig. S7b). This indicated that the stability of the sRNA plays an important role in silencing. The sRNAs *cpx*Qmut7 and 8 had a long region of complementarity to the 5’UTR(T7), with a ΔG_1_ of approximately − 40 kcal/mol. Nevertheless, the negative ΔG_1_-value did not compensate for the low stability of the secondary structure, with ΔG_2_-values of − 6.40 kcal/mol and − 6.20 kcal/mol, respectively. If the sRNA is immediately degraded, it cannot form a stable complex with the mRNA making the ΔG_1_-value of no relevance. A balance is needed between the sRNA secondary structure and complementarity to the 5’UTR(T7) in order to significantly improve production of the membrane-targeted protein sfGFP (Fig. 6d).

Finally, the sRNA-based stress circuit was applied for the overexpression of functional MPs. Two *E. coli* MPs were chosen: GarP and YidC. Microscopic visualization using the Airyscan technology, showed aggregation and membrane localization of GarP and YidC overexpression, respectively. Since GarP is a proven TM protein in *E. coli*, these aggregates were caused by the saturation of insertion machineries rather than the lack of recognition as a MP. YidC is mainly localized in the membrane with more proteins situated near the poles [80]. According to Urbanus et al. [81], the accumulation at the poles is localized in the membrane and not in the cytoplasm, as is the case with inclusion bodies. Possibly, GarP aggregated more quickly than YidC due to the number of TMDs, namely 12, compared to YidC, which contains six TMDs. The higher hydrophobicity of the protein is likely to increase the chance of hydrophobic interactions, which lead to the formation of aggregates. Without addition of the stress circuit, overexpression of both proteins severely hampered cell growth (Fig. 7b). Addition of the dynamically regulated stress circuit significantly increased cell growth in both cases and decreased protein production per cell (Fig. 7d). However, total protein production was not increased for GarP, even though cell growth was improved (Fig. 7b, c). The decreased GarP protein production suggested that the circuit responded to more than solely membrane stress. Many of the triggers of the Cpx-pathway are still unknown but CpxA-independent activation of CpxR has already been studied. There is a growing body of evidence that cytoplasmic signals such as the presence of certain salts, metals, the metabolic acetate pathway, decreased levels of cyclic AMP, among others, can trigger CpxR phosphorylation independent of CpxA. More specifically, it has been proposed that acetyl phosphate (acetyl-P), the intermediate of the phosphotransacetylase (Pta)-acetatekinase (AckA) pathway, can transfer its phosphoryl group to CpxR [40, 82]. This pathway is a result from the overflow metabolism which occurs in fast growing bacterial cells. At high growth rates, the *E. coli* cell switches from aerobic respiration to anaerobic fermentation. Despite the availability of oxygen, fermentation allows faster ATP production per unit membrane area and results in a lower proteome cost of energy biogenesis [83, 84]. The Pta-AckA pathway will not only generate ATP but also recycles CoA facilitating the glycolytic flux. In addition, other non-envelope associated stimuli of the Cpx pathway exist which could be correlated to heterologous protein overexpression. In the case of GarP, protein aggregate formation—before and after application of the stress circuit—was observed. GarP contains 12 TMDs probably causing hydrophobic interactions between un- and misfolded GarP proteins which resulted in the formation of protein aggregates in the crowded intracellular environment. The field of protein aggregation and their impact on reproduction and fitness remains a grey zone although recent work suggests that protein aggregation can have an effect on gene expression and cellular resource allocation [85]. One of these cell-survival strategies employs heat-shock proteins (HSPs), e.g., the HSP70 system, which also mediates refolding of unfolded or aggregated proteins by redirecting cellular resources towards several chaperones and proteases, e.g., the DnaKJE or GroESL chaperone systems [86–88]. A crucial component in all these responses is the heat-shock response σ-factor (σ^32^ or RpoH). RpoH is not only implicated in the regulation of several chaperone systems but also in the control of genes that are upregulated upon metabolic burden, e.g., IbpAB [89]. Several of these processes are connected to the CpxAR mechanism. For example, it is known that CpxR-P activates RpoH [77] or that the Cpx system can react to several stresses, e.g., aminoglycoside-caused stress, and this by activating several HSPs such as IbpAB [90]. In conclusion, P_*cpx*P(+5)_ is not only directly activated by membrane stress but can also indirectly be triggered by other imbalances and stress responses, such as several heat shock responses, caused by the overexpression of membrane proteins. For this reason, the stress source could be an important factor in the level of activation of P_*cpx*P(+5)_. More in-depth experiments, e.g., RNAseq or microarray analysis, are needed to study the sRNA levels in these cells and understand the reason for productivity decrease.

Although cell fitness did improve, by means of specific growth rate, microscopy results still indicated elongated cells. This shape plasticity is considered a survival strategy to cope with stressful conditions, e.g., DNA damage, antibiotic treatment, or host immune systems [91]. For this reason, we concluded that the dynamic stress circuit temporarily relieves the cell from additional stress which allows the cell to divide more frequently. Consequently, the designed IM stress circuit aims to decrease protein production per cell and hence stress levels which ultimately will allow the cells to grow and divide. As a result, protein numbers per cell decrease but on a culture level total protein production increases. Similar systems exist such as the *E. coli* BL21(DE3) strain or transcriptional feedback regulation leading to an optimal level of mRNA of the target MP [14, 21]. However, to our knowledge the designed membrane stress circuit is the first reported system using the *cpx*Q sRNA to dynamically regulate this stress in cell factories. Up to now, it was not possible to completely remove the stressful conditions connected to heterologous overexpression. More fundamental research is needed to understand the interaction of the Cpx pathway with other metabolic pathways in the cell. It is important to note that fundamental in-depth experiments such as RT-qPCR or sRNA-mRNA binding assays can be performed to further study off-target effects and interaction with the target mRNA. Moreover, to conclude general applicability of the stress circuit, expression of additional MPs, e.g., pro- and eukaryotic, are needed.

Finally, the choice of sRNA design can be an important tool to obtain a desirable output. The smart library of sRNAs pointed towards the importance of the sRNA design to obtain a significant effect on cell growth and productivity [89]. A stress source mainly originating from the membrane, was effective in both increasing cell fitness and total protein production and this in contrast to a cytoplasmic stress source which decreased productivity. As discussed, this could be correlated with the responsiveness of the stress-sensitive promoter to multiple triggers and hence a higher level of activation. The promiscuous effect of the promoter combined with the smart library offers the opportunity to design a sRNA which forms a less stable sRNA-mRNA complex which could finally result in higher productivities for the overexpression of almost all kinds of proteins. More specifically, we advise the implementation of the *cpx*Qmut3 variant for optimal and increased membrane protein production. This variant outperforms other *cpx*Qmut variants because of its optimal balance between sRNA-mRNA interaction (ΔG_1_-value of − 12.12) and sRNA stability (ΔG_2_-value of − 18.80). However, due to the promiscuity of the stress-sensitive promoter, other variants could be considered if one would opt for dynamic gene regulation of non-membrane proteins.

## Conclusions

A dynamically regulated feedback circuit was developed that can sense membrane-related toxicity signals and responds to it by balancing the metabolic state of the cell and more specifically by downregulating the expression of the MP of interest. This negative feedback mechanism was established using a simple-to-use genetic control element based on post-transcriptional regulation: small non-coding RNAs. The seed region of the native sRNA *cpx*Q was altered and optimized to bind to the 5’UTR upstream of our gene of interest. This strategy significantly increased the maximum specific growth rate and enhanced total protein production two- to three-fold for *E. coli* strains expressing membrane-targeted proteins. In contrast, if the protein accumulated in the cytoplasm as aggregates, the sRNA-based feedback control was still effective for improving cell growth but resulted in a decreased total protein production. This result suggests promiscuity of the sensor (P_*cpx*P(+5)_) for more than solely membrane stress.

## Material and methods

### Chemical, oligonucleotides and molecular biology

All the products were purchased from Sigma-Aldrich (Diegem, Belgium) unless stated otherwise. Agarose and ethidium bromide were purchased from Thermo Fisher Scientific (Erembodegem, Bel- gium). Standard molecular biology procedures were conducted as described by Sambrook et al. [92]. A variety of polymerases were used for the different types of PCR reactions: PrimeSTAR HS DNA polymerase (Takara, Westburg, Leusden, The Netherlands) and Q5 polymerase (New England Biolabs, County Road, Ipswich, MA, USA) were used for short DNA fragments (< 4 kb). PrimeSTAR GXL polymerase (Takara Bio, France) was used for long DNA fragments (> 4 kb). All DNA fragments were purified using the innuPREP PCR-pure Kit (Analytik Jena AG, Germany). All plasmids were isolated from bacterial cultures using the innuPREP Plasmid Mini Kit (Analytik Jena AG, Germany). Oligonucleotides were purchased from Integrated DNA Technologies (Leuven, Belgium). Sequencing services were conducted by Macrogen (Amsterdam, The Netherlands).

### Bacterial strains

*E. coli* One Shot Top10 Electrocomp™ (Invitrogen, Carlsbad, California, USA) were used for the construction and maintenance of all plasmids. *E. coli* DH10B was used to allow inducible expression with L-arabinose. For membrane protein expression, *E. coli* DE3 K12 MG1656Δ*end*AΔ*rec*A was used to allow inducible expression of the P_T7_ promoter using isopropyl β-D-1-thiogalactopyranoside (IPTG) as inducer. Expression vectors were introduced into their host cells by electroporation [92]. Bacterial strains that were used in this study were listed in Additional file 1: Table S2.

### Media and culture conditions

The culture medium lysogeny broth (LB) was used for cloning purposes. Transformed cultures were plated out on lysogenic broth agar (LBA) and grown overnight at 30 °C. If required, LB and LBA medium were supplemented with appropriate antibiotics (100 μg/mL ampicillin or 50 μg/ml kanamycin). For microscopy and the indirect analysis method, strains were grown in minimal medium glucose (MMGlc). If using DH10B cells, 0.4% casamino acids were added. If required, the culture medium was supplemented with appropriate antibiotics and inducers (0–1% L-arabinose or 0–0.2 mM IPTG). For fluorescence experiments with the smart library of sRNAs, strains were grown in MOPS EZ Rich Defined medium (Teknova) with 100 μg/mL ampicillin and 2% glucose as carbon source. For all fluorescent measurements, independent of medium (EZ Rich or MMGlc) used, precultures were grown overnight on a Compact Digital Microplate Shaker (Thermo Fisher Scientific) at 30 °C and 800 rpm, in 96-well flat bottomed microtiter plates (Greiner Bio-One), in 150 μL LB per well with appropriate antibiotics, enclosed by a Breathe-Easy sticker. Precultures were used for 1/150 dilution in 150 μL of defined medium (EZ Rich or MMGlc) containing a specific concentration of the inducer, and were grown for 24 h at 30 °C and 800 rpm unless stated otherwise.

### Plasmid construction

All plasmids for indirect inner-membrane (IM) stress analysis, microscopy (TMD-sfGFP fusion proteins), design evaluation (D1-D4), the smart sRNA library and overproduction of MPs were constructed using a combination of circular polymerase extension cloning (CPEC) and single strand assembly (SSA) [93]. These plasmids will be referred to as production plasmids. Characteristics of the corresponding plasmid backbones are listed in Additional file 1: Table S9. The genes *yid*C, *gar*P, *nlp*E, *soh*B(TMD) and *yhc*B(TMD) were PCR amplified using purified *E. coli* MG1655 genome (Genbank accession number U00096) as template. All proteins, used in this study, are listed in Additional file 1: Table S7. The rationally designed sRNA sequences and stress-sensitive promoter (P_*cpx*P(+5)_) were ordered as single stranded oligonucleotides from Integrated DNA Technologies. Part of the gene *cpx*P and P_*cpx*P_ promoter were PCR amplified using purified *E. coli* MG1655 genome as template. The *cpx*P 3’ end, containing *cpx*Qmut1, was added using CPEC. The corresponding plasmids are listed in Additional file 1: Table S6. More detailed plasmid maps are given in Additional file 1: Fig. S12. All DNA sequences (coding, promoter and 5’UTR) are listed in Additional file 1: Table S10.

Plasmids for small RNA engineering, were constructed using cloning plasmids (pCPs) and pBR322-based expression plasmids (pEX-BR322) as described in Coussement et al. [94]. To this end, two sets of pCPs, i.e., pCP(1–2) and pCP(2–3), were generated using CPEC. In short, each rationally designed sRNA sequence (*cpx*Qmut1-3) with inducible P_BAD_ promoter – and AraC for regulation—was cloned in a pCP(1–2) containing two consecutive BsaI cut sites (Golden Gate sites, or GG sites). Besides, a pCP(1–2) plasmid was subcloned with a truncated, non-functional, part of *sfgfp*. This truncated *sfgfp* (sfGFP(trunc)) is based on the *sfgfp* gene devoid of promoter, RBS and start codon [95]. The pCP(2–3) plasmid was subcloned with the P_T7_ promoter, RBS(T7) and *sfgfp* gene.

These pCPs were assembled into an expression plasmid (pEX) with a specific origin of replication (here pBR322) using Golden Gate assembly [96]. To use mKate2 expression for normalization of fluorescence data, the *pro*B promoter (P_*pro*B_) and mKate2 sequence were prior to the Golden Gate assembly subcloned on pEX-BR322 using CPEC. More detailed plasmid maps are given in Additional file 1: Fig. S12. All DNA sequences (coding, promoter and 5’UTR) are listed in Additional file 1: Table S10.

### In vivo fluorescence measurements

Strains, as three biological replicates (n = 3 for each IPTG or L-arabinose concentration), were prepared as described above. They were grown for 24 h at 30 °C and fluorescence and OD_600_ were continuously measured using a Tecan M200 infinite PRO with i-control software version 1.1. For measuring mKate2 fluorescence, excitation and emission wavelengths of 588 and 633 nm were used (gain 100), respectively. For measuring sfGFP fluorescence excitation and emission wavelengths of 488 and 510 nm were used (gain 70), respectively. Optical density was measured at a wavelength of 600 nm.

### Confocal light scanning microscopy

For microscopy analysis, Zeiss LSM 780 confocal scanning light microscopy, with Airyscan technology, was used. This service was provided by the Bio-imaging Facility at VIB Ghent. Microscopy was done using μ-slide 8 well coverslips (ibidi) coated with a 0.01% poly-L-lysine (molecular weight of 70–150 kDa) solution. Cell culture of interest was grown in 5 mL LB medium for 18 h and diluted 10 times in phosphate-buffered saline (PBS). Subsequently, 500 μL of this medium was dyed with 1 μL of the red FM4-64 membrane dye (1 mg/mL stock) (Thermo Fisher Scientific). The dyed cell culture was applied on the μ-slide 8 well coverslip and incubated for 30 min at room temperature. Next, the coverslip well was pipetted until dry and 2–3 droplets of mounting medium (1% n-propyl-gallate in glycerol) were added. The image processing package Fiji v1.8.0172, a distribution of ImageJ, was used to compose intensity profiles.

### Smart sRNA library construction

The RNA secondary structure and associated ΔG_2_-value was calculated using RNAfold. The interaction of the designed sRNA with 5’UTR(T7) and associated ΔG_1_-value was calculated using RNAcofold [57].

### Data and statistical analysis

Data were analysed using pandas (www.pandas.pydata.org) unless stated otherwise. Maximal growth rates were determined by plotting $\mathrm{ln}\frac{{({OD}_{600})}_{t}}{{({OD}_{600})}_{{t}_{0}}}$ values as a function of time and fitting Richards growth model. Pairwise comparisons between different strains were done by a two-sided t-test using the scipy.stats package in Python. One-way ANOVA was performed using the scipy.stats package in Python. Linear regression was performed using the statsmodels.anova package in Python. In all cases, a significance level of 0.05 was applied.

For fluorescence measurements, defined medium (MM or MOPS EZ Rich medium) without cell culture was used to correct for background fluorescence and optical density of the medium (FP_med_ and OD_med_, respectively) for each imposed inducer concentration. To account for background fluorescence and optical density (FP_Blank_ and OD_Blank_, respectively) of the cell culture, *E. coli* TOP10, DE3 or DH10B cells without plasmid, were used. Thus, the corrected fluorescence, normalized for optical density, was calculated as follows:

$$\left( {\frac{FP}{{OD}}} \right)_{cor} = \frac{{FP - FP_{med} }}{{OD - OD_{med} }} - \frac{{FP_{Blank} - FP_{med} }}{{OD_{Blank} - OD_{med} }}$$

## Supplementary Information

**Additional file 1**: **Figure S1**. Growth curves corresponding to indirect stress detection tool. **Figure S2**. *cpx*Q binding sites for *nha*B mRNA. **Figure S3**. Bar plots representing the output sfGFP/OD_600_, mKate2, sfGFP and µ_max_ for the control plasmid (blue) and the plasmid expressing the sRNA *cpx*Qmut1 (grey) and this for different L-arabinose concentrations. **Figure S4**. Confocal scanning light microscopy images with intensity cross-section profiles for both the red FM4-64 membrane dye (purple colour) and the green fluorescent protein signal of TMD-sfGFP fusion proteins. **Figure S5**. Evaluation of the three dual plasmid designs (D1-3) on growth and protein production of the membrane targeted sfGFP. **Figure S6**. Library of nine different *cpx*Q mutants in their secondary structure with the associated ΔG_2_ value. **Figure S7**. Effect of the smart library of sRNA-based stress circuits on fluorescence intensity. **Figure S8**. Relative mKate2/OD_600_ fluorescence of *Escherichia coli* (*E. coli*) DE3 cells producing either sfGFP (cytoplasmatic superfolder green fluorescent protein, negative control), NlpE (IMP of *E. coli*, positive control), SohB(TMD)-sfGFP, YidC-sfGFP and GarP-sfGFP for several IPTG-concentrations (Timepoint: 10h, stationary phase). **Figure S9**. Effect of the sRNA-based circuit, respectively with *cpx*Qmut1 (Stress Circuit 1) and *cpx*Qmut3 (Stress Circuit 3), on cell growth and fluorescence intensity. **Figure S10**. Confocal scanning light microscopy images with intensity cross-section profiles for both the red FM4-64 membrane dye (purple colour) and the green fluorescent protein signal of MPs YidC and GarP (sfGFP, green colour). **Figure S11**. Effect of the non-specific and specific sRNA-based stress circuit on protein production of functional MPs expression. **Figure S12**. Detailed plasmid maps of the vectors created in this work. **Table S1**. List of fold changes for NlpE and sfGFP expression (at OD_600_ = 0.3) measured for indirect IM stress analysis. **Table S2**. Bacterial *Escherichia coli *strains that were used in this study. **Table S3**. List of DNA sequences used in this study to design the smart small RNA library. **Table S4**. Two sample t-tests performed in this study. **Table S5**. Statistical one-way ANOVA performed in this study. **Table S6**. Plasmids that were used and constructed throughout this study. **Table S7**. List of proteins used in this study for membrane localisation and membrane targeting. **Table S8**. List of fold changes for NlpE, sfGFP, SohB(TMD)-sfGFP, YidC-sfGFP and GarP-sfGFP expression (at t = 20h) measured for indirect IM stress analysis. **Table S9**. Overview of the different plasmid backbones used in this study and their assigned function. **Table S10**. List of DNA sequences used in this study (coding sequences, promoter, 5’UTRs and terminator sequences).

## Data Availability

All data generated or analyzed during this study are included in this published article [and its Additional file 1].
