# Supplementary material for: Dynamic feedback regulation for efficient membrane protein production using a small RNA-based genetic circuit in Escherichia coli
Source: Microb Cell Fact. 2022 Dec 15;21:260. doi: 10.1186/s12934-022-01983-2 (PMC9753035; doi:10.1186/s12934-022-01983-2)
Supplement: Supplementary file 1 — Additional file 1: Figure S1. Growth curves corresponding to indirect stress detection tool. Figure S2. cpxQ binding sites for nhaB mRNA. Figure S3. Bar plots representing the output sfGFP/OD600, mKate2, sfGFP and µmax for the control plasmid (blue) and the plasmid expressing the sRNA cpxQmut1 (grey) and this for different L-arabinose concentrations. Figure S4. Confocal scanning light microscopy images with intensity cross-section profiles for both the red FM4-64 membrane dye (purple colour) and the green fluorescent protein signal of TMD-sfGFP fusion proteins. Figure S5. Evaluation of the three dual plasmid designs (D1-3) on growth and protein production of the membrane targeted sfGFP. Figure S6. Library of nine different cpxQ mutants in their secondary structure with the associated ΔG2 value. Figure S7. Effect of the smart library of sRNA-based stress circuits on fluorescence intensity. Figure S8. Relative mKate2/OD600 fluorescence of Escherichia coli (E. coli) DE3 cells producing either sfGFP (cytoplasmatic superfolder green fluorescent protein, negative control), NlpE (IMP of E. coli, positive control), SohB(TMD)-sfGFP, YidC-sfGFP and GarP-sfGFP for several IPTG-concentrations (Timepoint: 10h, stationary phase). Figure S9. Effect of the sRNA-based circuit, respectively with cpxQmut1 (Stress Circuit 1) and cpxQmut3 (Stress Circuit 3), on cell growth and fluorescence intensity. Figure S10. Confocal scanning light microscopy images with intensity cross-section profiles for both the red FM4-64 membrane dye (purple colour) and the green fluorescent protein signal of MPs YidC and GarP (sfGFP, green colour). Figure S11. Effect of the non-specific and specific sRNA-based stress circuit on protein production of functional MPs expression. Figure S12. Detailed plasmid maps of the vectors created in this work. Table S1. List of fold changes for NlpE and sfGFP expression (at OD600 = 0.3) measured for indirect IM stress analysis. Table S2. Bacterial Escherichia coli strains t [file 12934_2022_1983_MOESM1_ESM.docx]

Additional file

Dynamic feedback regulation for efficient membrane protein production using a small RNA-based genetic circuit in *Escherichia coli*

*Chiara Guidi, Lien De Wannemaeker, Jasmine De Baets, Wouter Demeester, Jo Maertens, Brecht De Paepe, & Marjan De Mey**

*Centre for Synthetic Biology (CSB), Ghent University, 9000 Ghent, Belgium*

**Corresponding author:*

*M. De Mey*

*Coupure links 653, 9000 Ghent, Belgium*

marjan.demey@ugent.be

*Tel: +32 9 2646028*

**Key words:**

Membrane proteins; *Escherichia coli*; sRNA-based genetic circuitry

**Overview**

**Additional file 1: Figure S1.** Growth curves corresponding to indirect stress detection tool.

**Additional file 1: Figure S2.** *cpx*Q binding sites for *nha*B mRNA.

**Additional file 1: Figure S3.** Bar plots representing the output sfGFP/OD_600_, mKate2, sfGFP and µ_max_ for the control plasmid (blue) and the plasmid expressing the sRNA *cpx*Qmut1 (grey) and this for different L-arabinose concentrations

**Additional file 1: Figure S4.** Confocal scanning light microscopy images with intensity cross-section profiles for both the red FM4-64 membrane dye (purple colour) and the green fluorescent protein signal of TMD-sfGFP fusion proteins.

**Additional file 1: Figure S5.** Evaluation of the three dual plasmid designs (D1-3) on growth and protein production of the membrane targeted sfGFP.

**Additional file 1: Figure S6.** Library of nine different *cpx*Q mutants in their secondary structure with the associated ΔG_2_ value.

**Additional file 1: Figure S7.** Effect of the smart library of sRNA-based stress circuits on fluorescence intensity.

**Additional file 1: Figure S8.** Relative mKate2/OD_600_ fluorescence of *Escherichia coli* (*E. coli*) DE3 cells (See Supplementary Table S1 for more details) producing either sfGFP (cytoplasmatic superfolder green fluorescent protein, negative control), NlpE (IMP of *E. coli*, positive control), SohB(TMD)-sfGFP, YidC-sfGFP and GarP-sfGFP for several IPTG-concentrations (Timepoint: 10h, stationary phase).

**Additional file 1: Figure S9.** Effect of the sRNA-based circuit, respectively with *cpx*Qmut1 (Stress Circuit 1) and *cpx*Qmut3 (Stress Circuit 3), on cell growth and fluorescence intensity.

**Additional file 1: Figure S10.** Confocal scanning light microscopy images with intensity cross-section profiles for both the red FM4-64 membrane dye (purple colour) and the green fluorescent protein signal of MPs YidC and GarP (sfGFP, green colour).

**Additional file 1: Figure S11.** Effect of the non-specific and specific sRNA-based stress circuit on protein production of functional MPs expression.

**Additional file 1: Figure S12.** Detailed plasmid maps of the vectors created in this work

**Additional file 1: Table S1.** List of fold changes for NlpE and sfGFP expression (at OD_600_ = 0.3) measured for indirect IM stress analysis.

**Additional file 1: Table S2.** Bacterial *Escherichia coli* strains that were used in this study.

**Additional file 1: Table S3.** List of DNA sequences used in this study to design the smart small RNA library.

**Additional file 1: Table S4.** Two sample t-tests performed in this study.

**Additional file 1: Table S5.** Statistical one-way ANOVA performed in this study.

**Additional file 1: Table S6.** Plasmids that were used and constructed throughout this study.

**Additional file 1: Table S7.** List of proteins used in this study for membrane localisation and membrane targeting.

**Additional file 1: Table S8.** List of fold changes for NlpE, sfGFP, SohB(TMD)-sfGFP, YidC-sfGFP and GarP-sfGFP expression (at t = 20h) measured for indirect IM stress analysis.

**Additional file 1: Table S9.** Overview of the different plasmid backbones used in this study and their assigned function.

**Additional file 1: Table S10.** List of DNA sequences used in this study (coding sequences, promoter, 5’UTRs and terminator sequences).


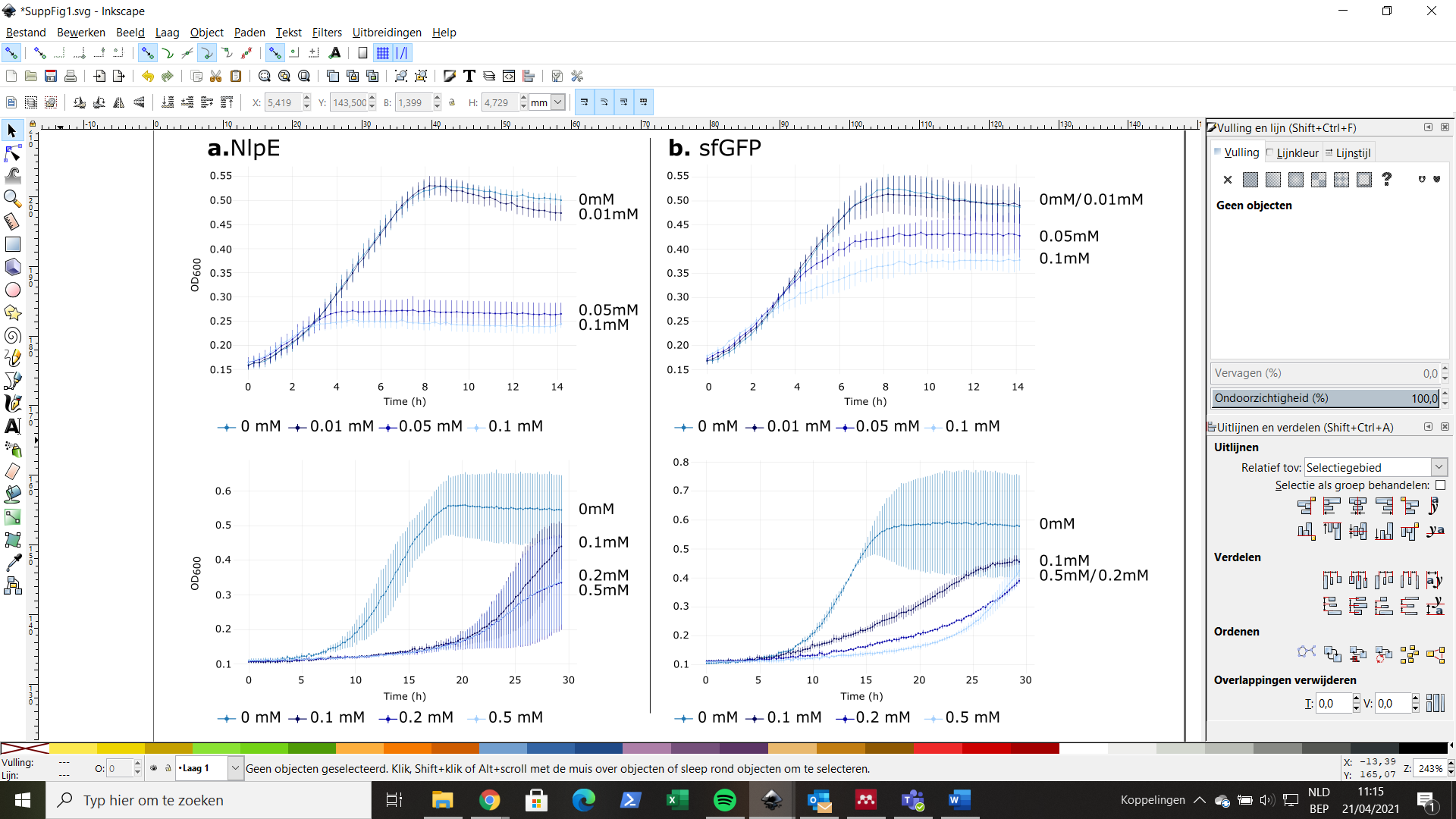
**Supplementary Figures**

**Supplementary Figure S1**. Growth curves corresponding to indirect stress detection tool. Detection circuit was evaluated for control proteins NlpE (a) and sfGFP (b), at an IPTG range from 0 to 0.1 mM (top) and from 0 to 0.5 mM (bottom) added post-lag phase. Error bars represent one standard deviation from the plotted mean value. IPTG = isopropyl β-D-thiogalactopyranoside, NlpE = lipoprotein in *Escherichia coli* (*E. coli*), OD_600_ = optical density measured at 600 nm, sfGFP = superfolder green fluorescent protein


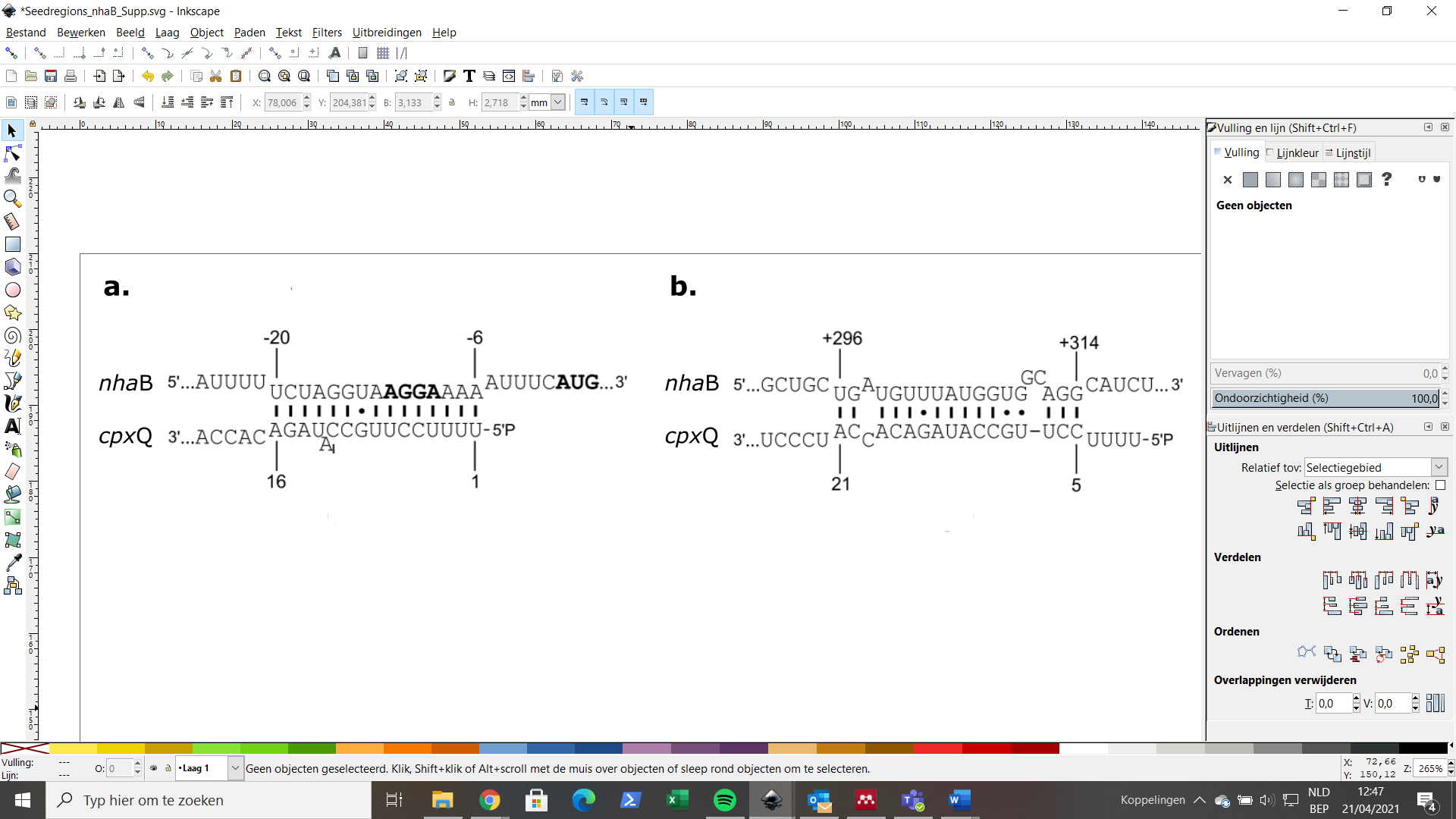


**Supplementary Figure S2.** *cpx*Q binding sites for *nha*B mRNA. (a) *cpx*Q-binding site for the TIR of *nha*B mRNA. (b) *cpx*Q-binding site for part of the coding sequence of *nha*B (1). mRNA = messenger RNA, TIR = translation initiation region, NhaB = sodium-proton anti-porter in *Escherichia coli*


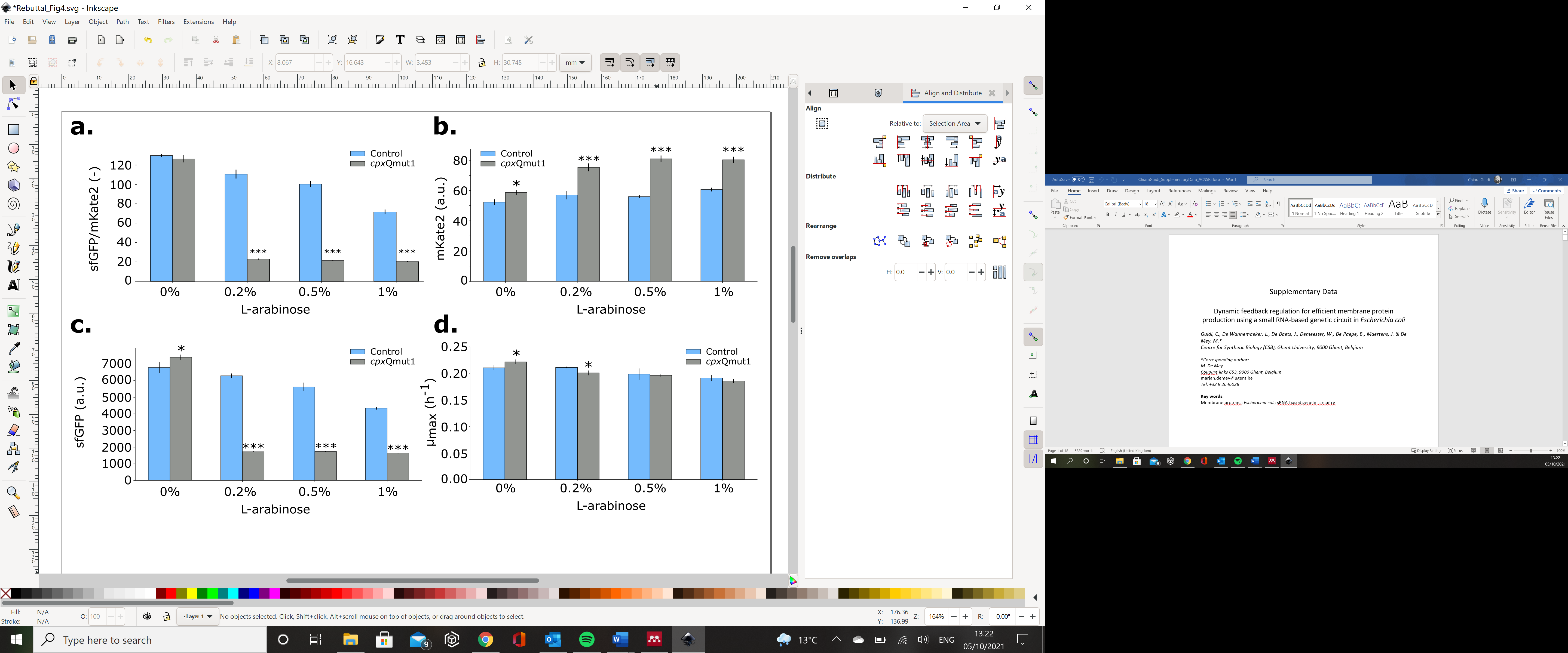


**Supplementary Figure S3.** (a) Bar plots representing the output sfGFP/mKate2 for the control plasmid (blue) and the plasmid expressing the sRNA *cpx*Qmut1 (grey) and this for different L-arabinose concentrations. (b) Bar plots representing the output mKate2 for the control plasmid (blue) and the plasmid expressing the sRNA *cpx*Qmut1 (grey) and this for different L-arabinose concentrations. (c) Bar plots representing the output sfGFP for the control plasmid (blue) and the plasmid expressing the sRNA *cpx*Qmut1 (grey) and this for different L-arabinose concentrations. (d) Specific growth rate (µ_max_) for strains (Escherichia coli DH10B) expressing the control plasmid (blue) or the plasmid expressing the sRNA cpxQmut1 and this for different L-arabinose concentrations. sfGFP/OD_600_, mKate2 and sfGFP values for an equal number (OD_600_ = 0.4, mid-exponential phase) of *Escherichia coli* DH10B cells were plotted. All experiments were carried out in replica triplicates (biological variation) and the error bars represent one standard deviation from the mean value. * = p-value < 0.05, *** = p-value < 0.001 obtained by conducting a two-sample t-test between strains expressing the control plasmid and strain expressing the sRNA *cpx*Qmut1 and this for each L-arabinose concentration.


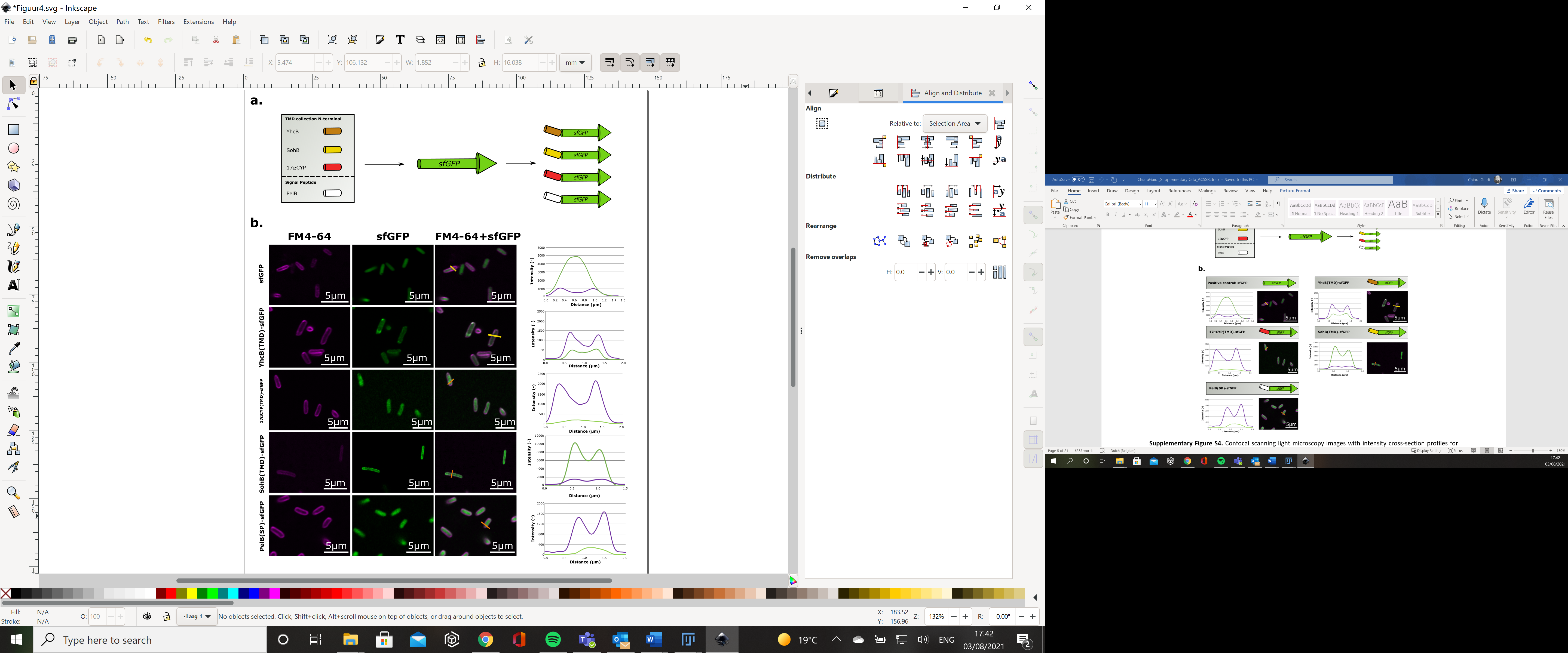
**Supplementary Figure S4.** Confocal scanning light microscopy images with intensity cross-section profiles for both the red FM4-64 membrane dye (purple colour) and the green fluorescent protein signal of TMD-sfGFP fusion proteins. ). Yellow bars represent the position used to make a cross-section profile. (a) Schematic representation of the TMD-sfGFP fusion strategy to investigate the effect of TMD amino acid sequence on membrane localisation in *Escherichia coli* (*E. coli*). (b) Confocal light scanning microscopy images with intensity cross-section profiles for both the red FM4-64 membrane dye (purple colour) and the green fluorescent protein signal (GFP, green colour). 17αCYP(TMD) = TMD of the bovine 17-α-hydroxylase, PelB = signal peptide of the pectate lyase B, sfGFP = superfolder green fluorescent protein, SohB = inner-membrane protein from *E. coli*, TMD = transmembrane domain, YhcB = putative subunit of a membrane-associated protein complex from *E. coli*


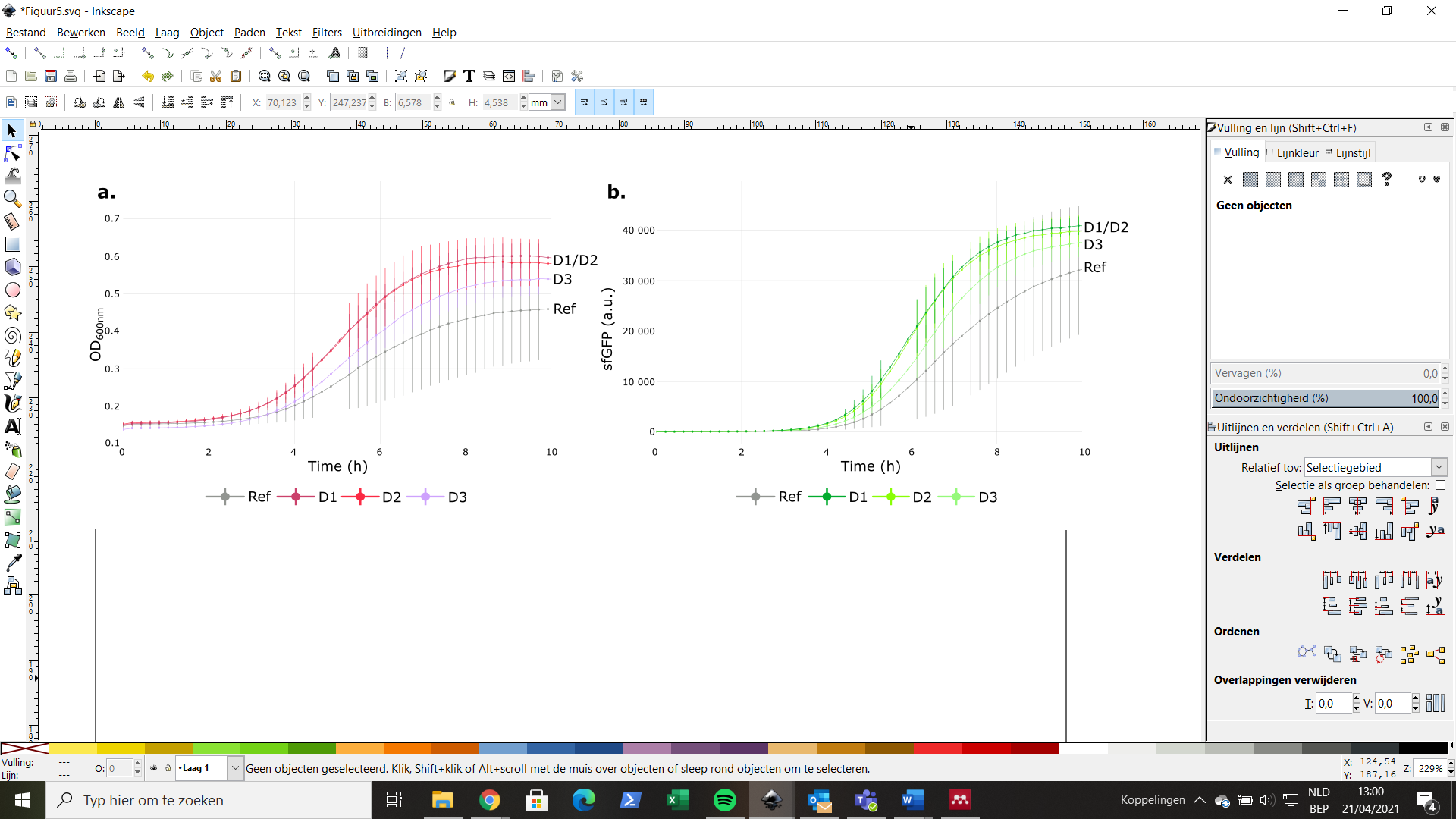
**Supplementary Figure S5.** Evaluation of the three dual plasmid designs (D1-3) on growth and protein production of the membrane targeted sfGFP. As reference the *Escherichia coli* MG1655 DE3 strain carrying SohB(TMD)-sfGFP (Ref) was used. (a) Growth curves (OD_600_) of strains Ref, DE3 + SohB(TMD)-sfGFP + P*_cpxP(+5)_*-cpxQmut1 (D1), DE3 + SohB(TMD)-sfGFP + P*_cpxP_*-cpxQmut1 (D2) and DE3 + SohB(TMD)-sfGFP + P*_cpxP_-*cpxP-cpxQmut1 (D3). (b) sfGFP intensity of the same strains measured in time. Inducer IPTG was added at the beginning, concentration = 0.1 mM. a.u. = arbitrary unit, OD_600_ = optical density measured at 600 nm, IPTG = isopropyl β-D-1-thiogalactopyranoside, TMD = transmembrane domain, SohB = inner-membrane protein from *Escherichia coli*, TMD = transmembrane domain


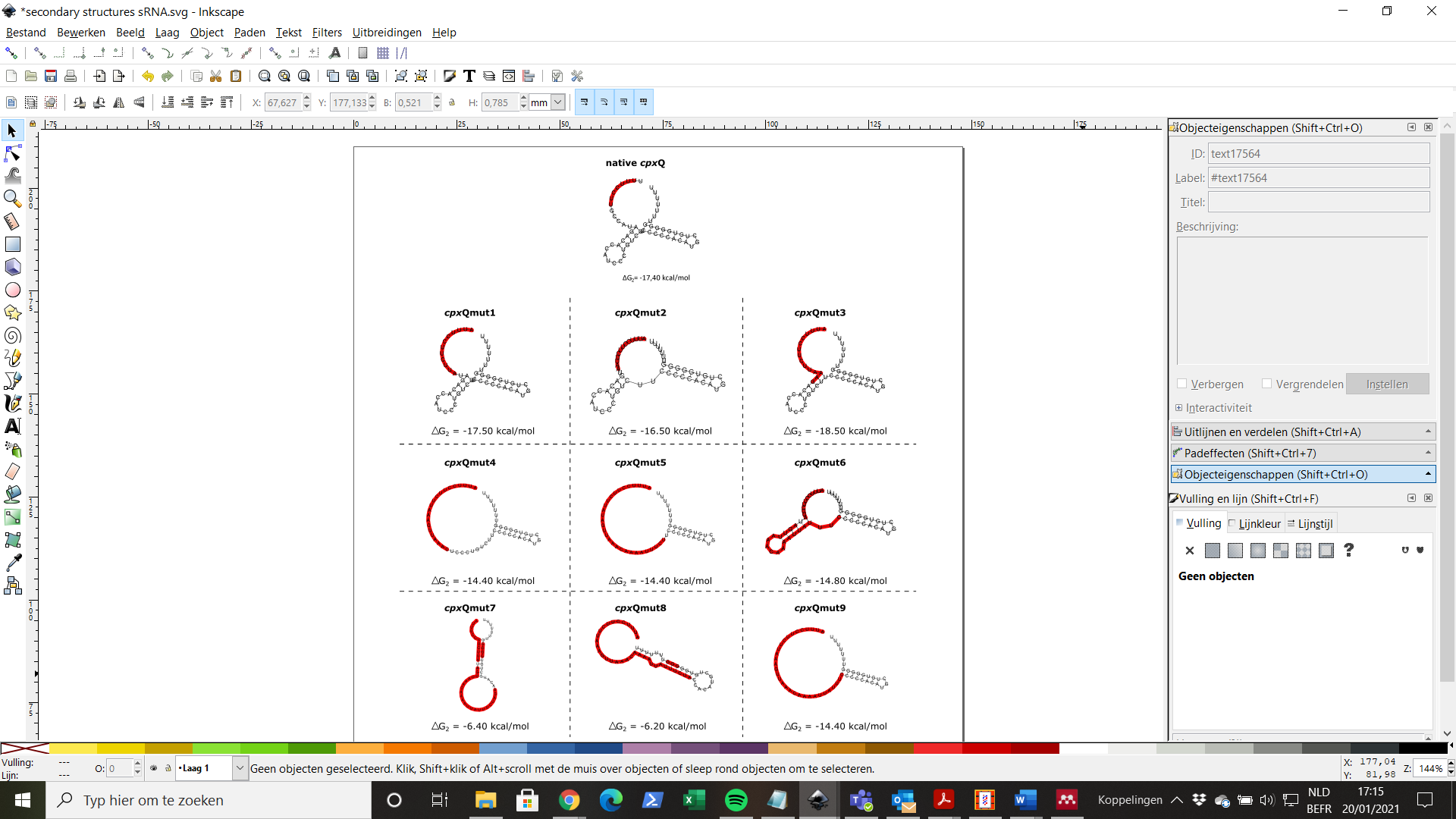
**Supplementary Figure S6**. Library of nine different *cpx*Q mutants in their secondary structure with the associated ΔG_2_ value, determined with the RNAfold tool of the ViennaRNA package. The nucleotides that bind to the TIR in the sRNA-mRNA complex are highlighted in red. ΔG_2_ = Gibbs free energy of the RNA secondary structure, TIR = translation initiation region


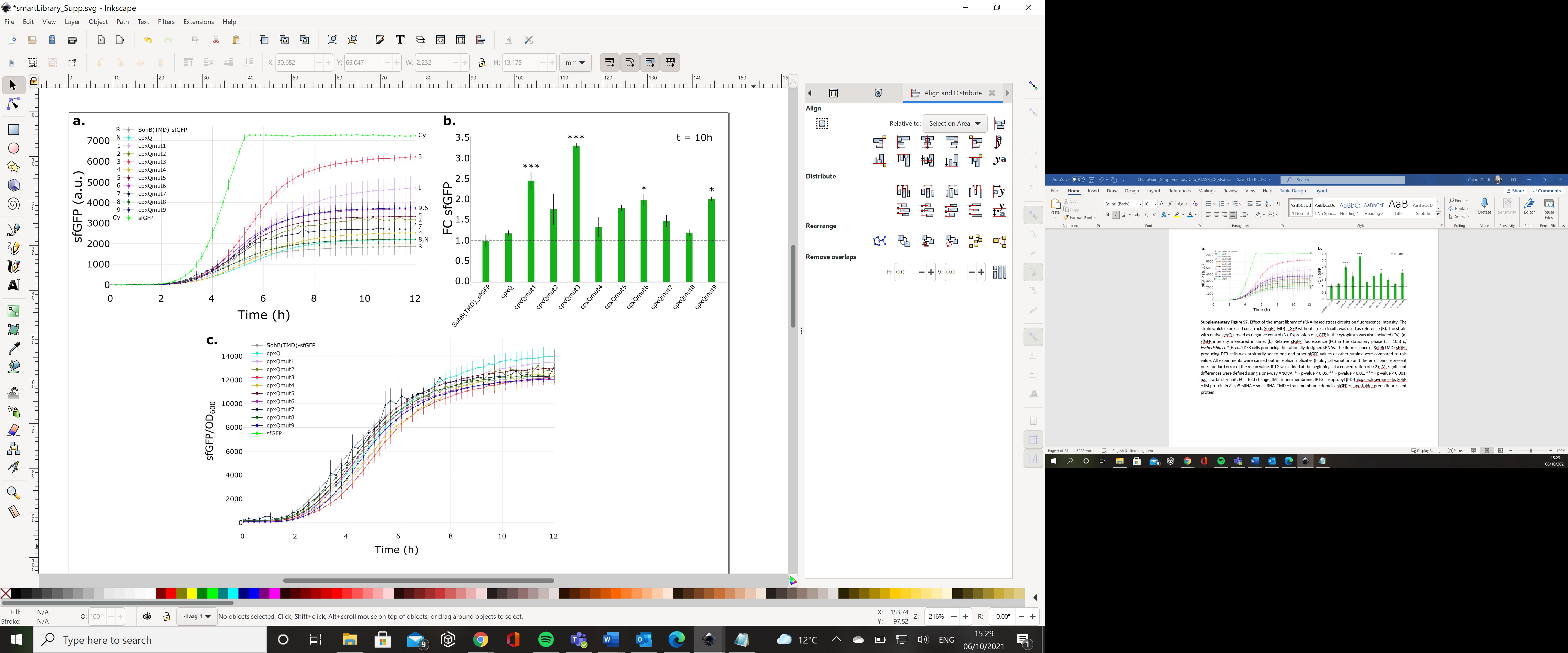


**Supplementary Figure S7.** Effect of the smart library of sRNA-based stress circuits on fluorescence intensity. The strain which expressed constructs SohB(TMD)-sfGFP without stress circuit, was used as reference (R). The strain with native *cpx*Q served as negative control (N). Expression of sfGFP in the cytoplasm was also included (Cy). (a) sfGFP intensity measured in time. (b) Relative sfGFP fluorescence (FC) in the stationary phase (t = 10h) *of Escherichia coli* (*E. coli*) DE3 cells producing the rationally designed sRNAs. The fluorescence of SohB(TMD)-sfGFP producing DE3 cells was arbitrarily set to one and other sfGFP values of other strains were compared to this value. (c) Ratio sfGFP/OD_600_ measured in time. This ratio was corrected for background fluorescence and optical density of the medium and the cell culture (*Escherichia coli* MG1656 DE3). All experiments were carried out in replica triplicates (biological variation) and the error bars represent one standard error of the mean value. IPTG was added at the beginning, at a concentration of 0.2 mM. Significant differences were defined using a one-way ANOVA. * = p-value < 0.05, *** = p-value < 0.001, a.u. = arbitrary unit, FC = fold change, IM = inner-membrane, IPTG = isopropyl β-D-thiogalactopyranoside, SohB = IM protein in *E. coli*, sRNA = small RNA, TMD = transmembrane domain, sfGFP = superfolder green fluorescent protein


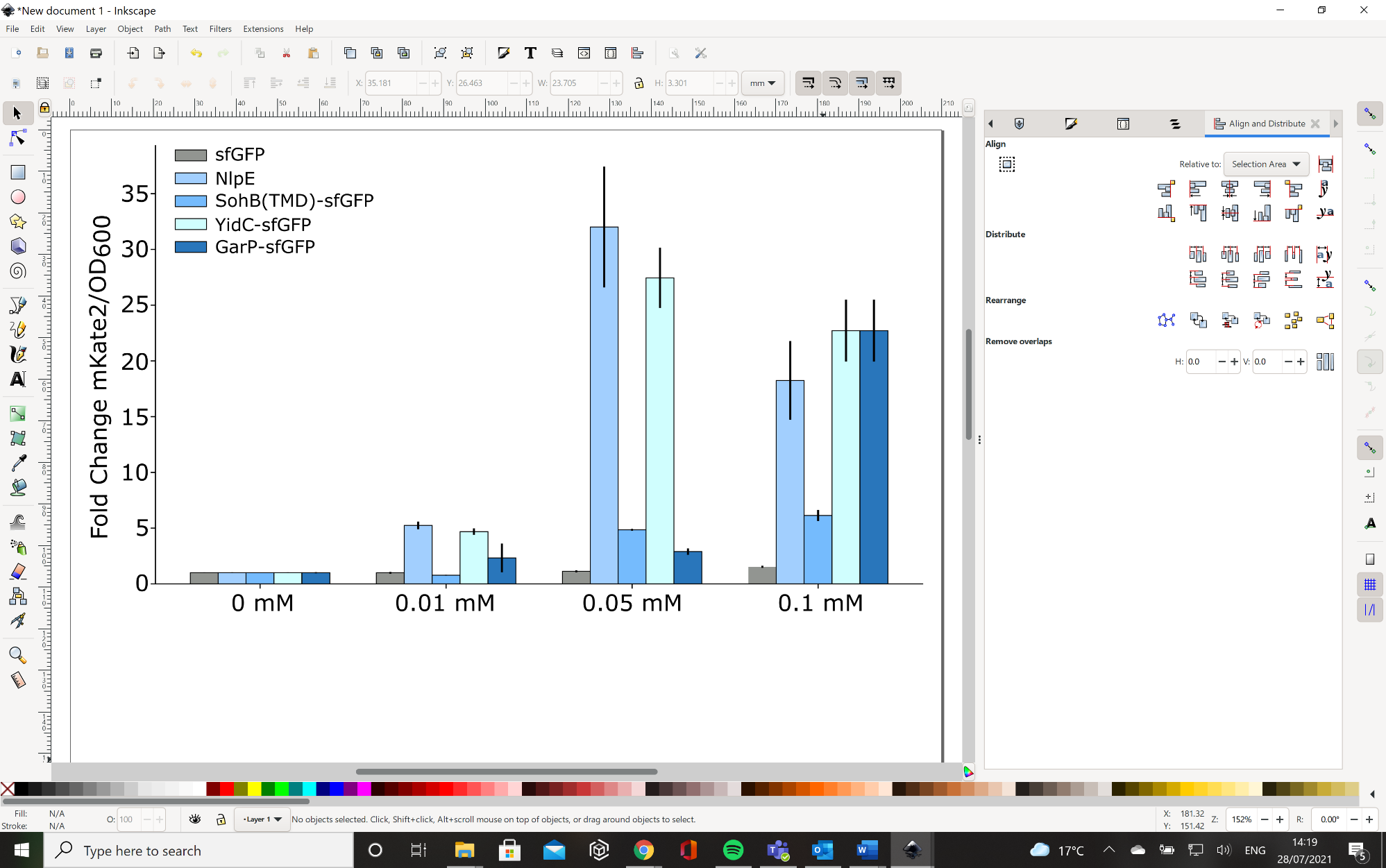
**Supplementary Figure S8.** Relative mKate2/OD_600_ fluorescence of *Escherichia coli* (*E. coli*) DE3 cells (See Supplementary Table S1 for more details) producing either sfGFP (cytoplasmatic superfolder green fluorescent protein, negative control), NlpE (IMP of *E. coli*, positive control), SohB(TMD)-sfGFP, YidC-sfGFP and GarP-sfGFP with membrane stress sensor for several IPTG-concentrations (Timepoint: 10h, stationary phase). The fluorescence of NlpE, sfGFP, SohB(TMD)-sfGFP, YidC-sfGFP and GarP-sfGFP producing DE3 cells at 0 mM IPTG is arbitrarily set to one and fluorescence of other IPTG concentrations was compared to this value. IM = inner-membrane, IMP = inner-membrane protein, mKate2 = red fluorescent protein. IPTG = isopropyl β-D-1-thiogalactopyranoside, TMD = transmembrane domain, SohB = inner-membrane protein from *E. coli,* YidC = membrane protein insertase in *E. coli*, GarP = TM transporter in *E. coli*, MP = membrane protein,


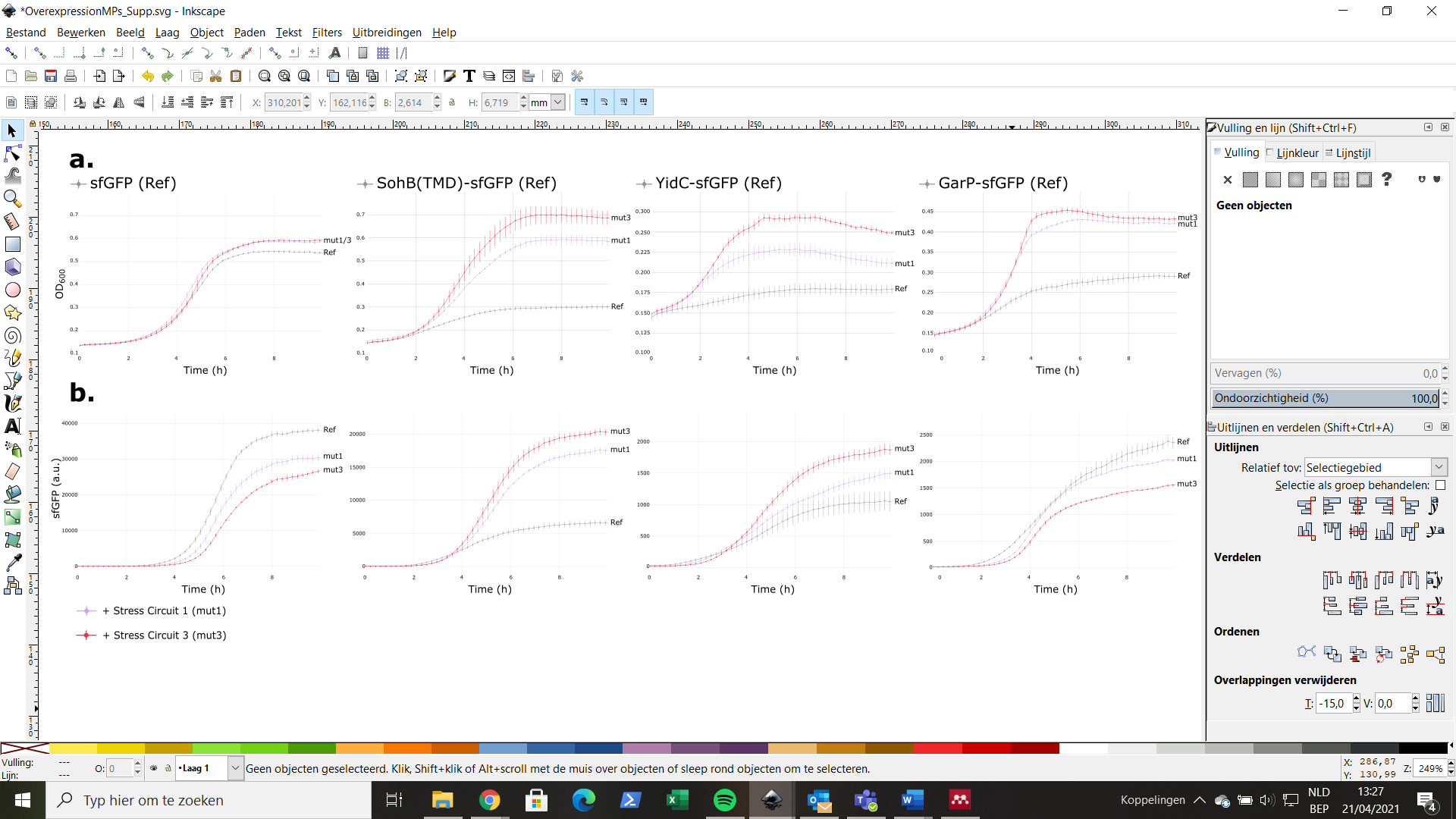
**Supplementary Figure S9.** Effect of the sRNA-based circuit, respectively with *cpx*Qmut1 (Stress Circuit 1) and *cpx*Qmut3 (Stress Circuit 3), on cell growth and fluorescence intensity. IM stress was sensed by promoter P*_cpx_*_P(+5)_ which activated sRNA expression. (a) Growth curves (OD_600_) for strains with and without circuits, measured in time. (b) sfGFP intensity, for strains with and without circuits, measured in time. Inducer IPTG was added at the beginning, concentration = 0.2 mM. a.u. = arbitrary unit, OD_600_ = optical density measured at 600 nm, IPTG = isopropyl β-D-1-thiogalactopyranoside, TMD = transmembrane domain, SohB = inner-membrane protein from *Escherichia coli*, Stress Circuit 1 = strain expressing MP and sRNA *cpx*Qmut1, Stress Circuit 3 = strain expressing MP and sRNA *cpx*Qmut3, MP = membrane protein, sRNA = small RNA


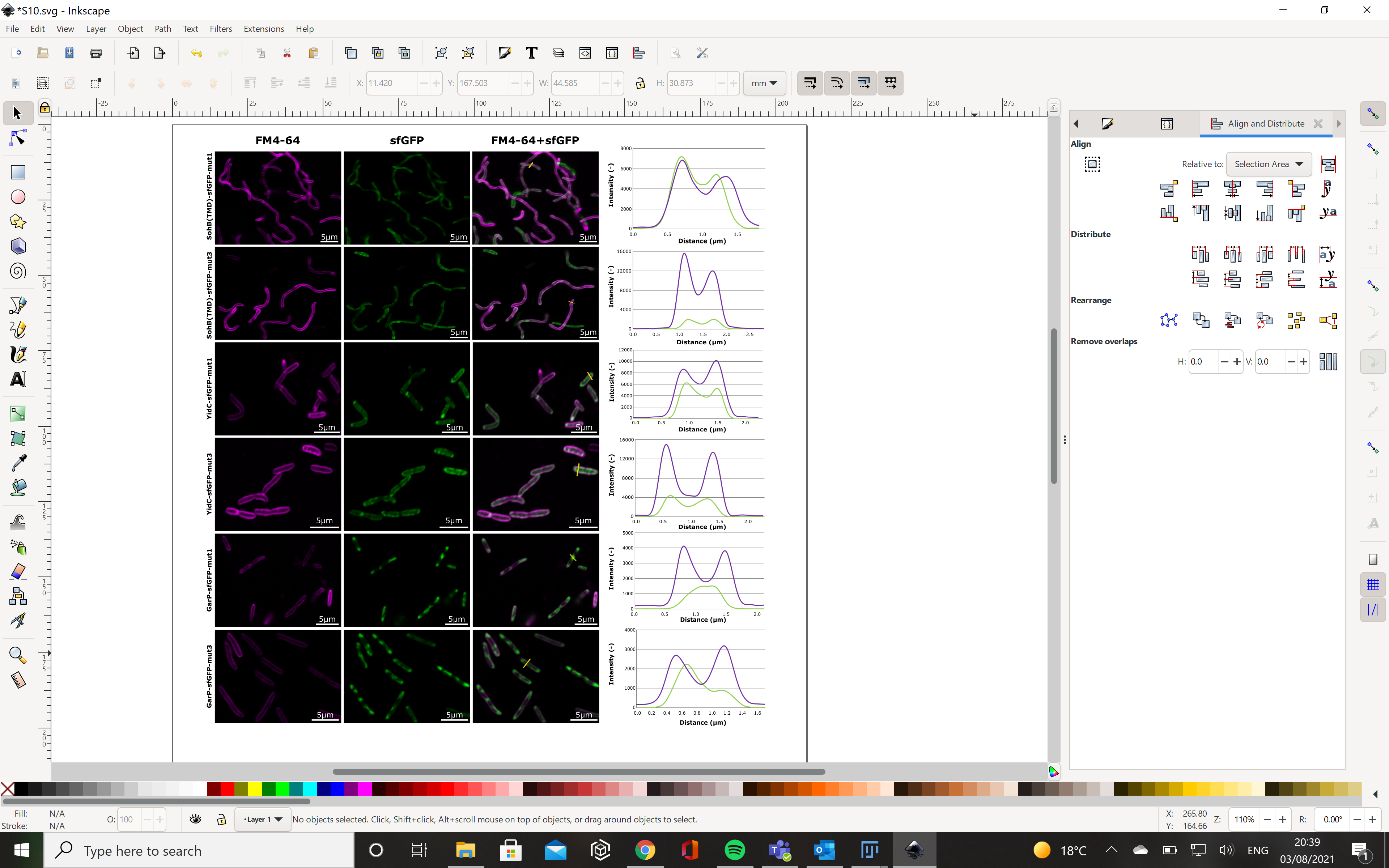


**Supplementary Figure S10**. Confocal scanning light microscopy images with intensity cross-section profiles for both the red FM4-64 membrane dye (purple colour) and the green fluorescent protein signal of MPs YidC and GarP (sfGFP, green colour). Yellow bars represent the position used to make a cross-section profile. Effect of both stress circuits on cellular localisation of SohB(TMD)-sfGFP, YidC-sfGFP and GarP-sfGFP. IPTG (0.2 mM) was added to induce protein expression. YidC = membrane protein insertase in *Escherichia coli* (*E. coli*), GarP = TM transporter in *E. coli*, MP = membrane protein, sfGFP = superfolder green fluorescent protein, SohB = inner-membrane protein from *E. coli*, Stress circuit 1 (mut1) = strain expressing MP and sRNA *cpx*Qmut1, Stress circuit 3 (mut3) = strain expressing MP and sRNA *cpx*Qmut3, TMD = transmembrane domain


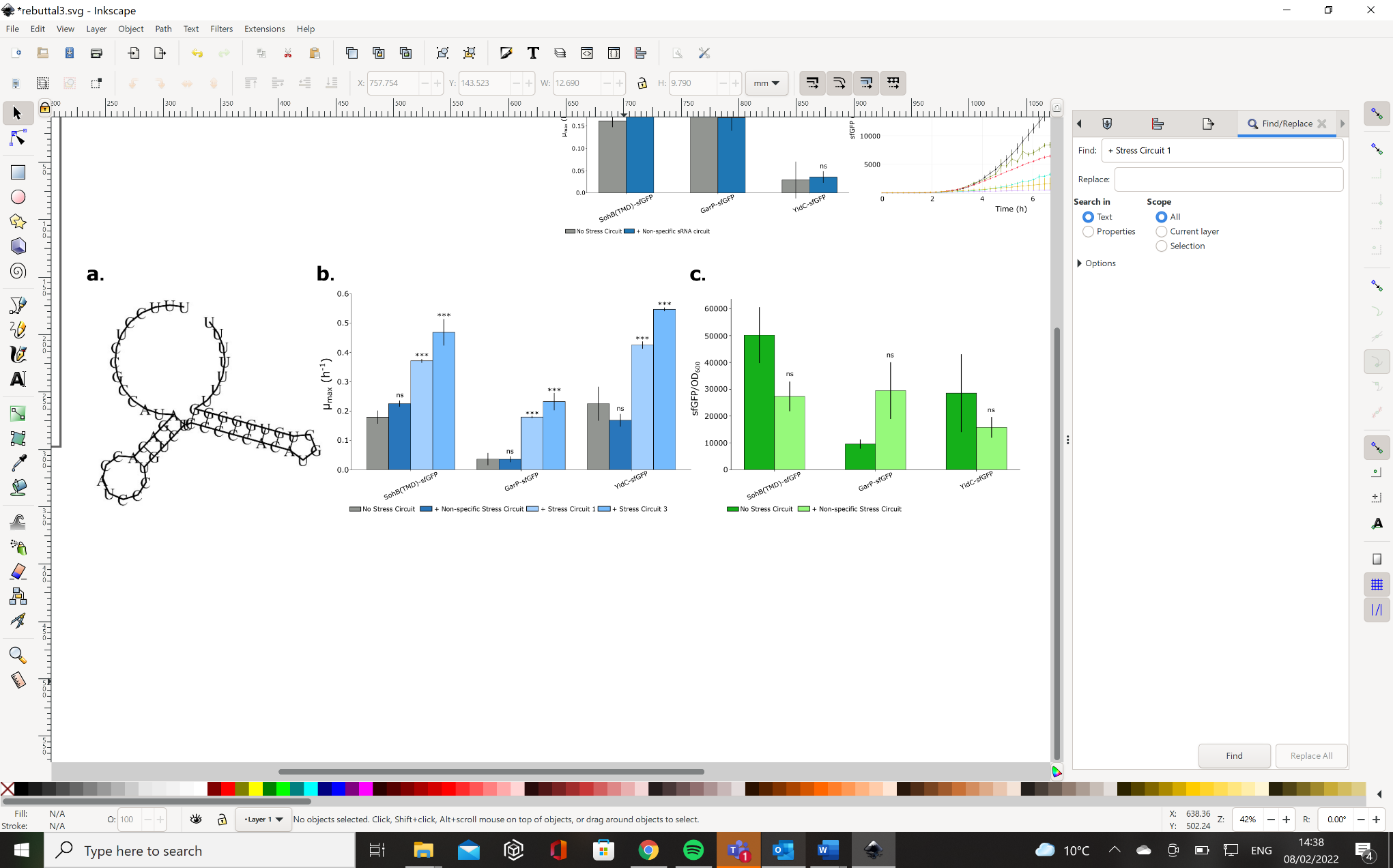


**Supplementary Figure S11.** Effect of the non-specific and specific sRNA-based stress circuit on protein production of functional MPs expression. (a) Secondary structure of the non-specific sRNA *cpx*Qmut. (b) Specific growth rate (µ_max_) for strains (*Escherichia coli* (*E. coli*) MG1656 DE3, Supplementary Table S1) expressing SohB(TMD)-sfGFP, YidC-sfGFP and GarP-sfGFP. (c) sfGFP/OD_600_-values for overexpression of proteins, SohB(TMD)-sfGFP, YidC-sfGFP and GarP-sfGFP without and with non-specific stress circuit. sfGFP/OD_600_ results for an equal number (OD_600_ = 0.15 for SohB(TMD)-sfGFP, mid exponential phase, OD_600_ = 0.2 for GarP-sfGFP, late exponential phase, OD_600_ = 0.14 for YidC-sfGFP, stationary phase) of *E. coli* MG1656 DE3 cells were plotted. This ratio was corrected for background fluorescence and optical density of the medium and the cell culture (*Escherichia coli* MG1656 DE3). All experiments were carried out in triplicates (biological variation) and the error bars represent the standard deviation from the mean value. Statistical difference between strains expressing the MP without stress circuit and with the non-specific stress circuit, was calculated using a two-sample t-test. Ns = non-significant, *** = p-value < 0.001, IPTG (0.2 mM) was added to induce protein expression. YidC = membrane protein insertase in *E. coli*, GarP = TM transporter in *E. coli*, MP = membrane protein, sfGFP = superfolder green fluorescent protein, SohB(TMD)= transmembrane domain of inner-membrane protein SohB from *E. coli*, Non-specific sRNA circuit = strain expressing MP and a non-specific sRNA


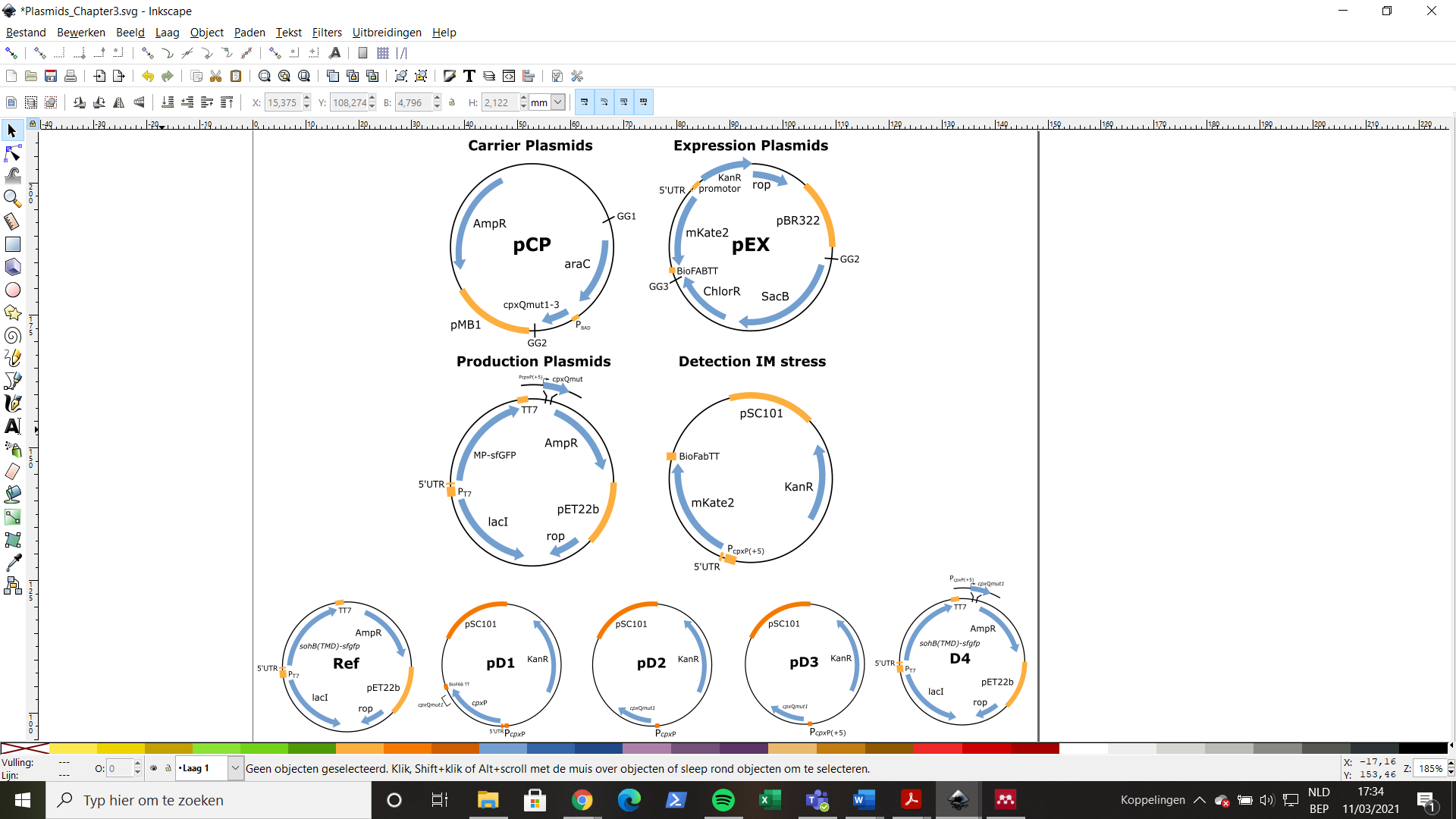
**Supplementary Figure S12.** Detailed plasmid maps of the vectors created in this work. Orange bars indicate functional regions such as origins of replication, promoters, 5’ UTRs or terminators. Blue arrows show particular coding sequences. The position for introduction of the smart sRNA library is indicated on the production plasmid. This plasmid served as the vector for expression of all MPs, fused to sfGFP, in this study. The three designs and reference strain for optimising of membrane-targeted sfGFP production are combined as follows: D1 = Ref + pD1, D2 = Ref + pD2, D3 = Ref + pD3, Amp = ampicillin, Chlor = chloramphenicol, GG = golden gate site, Kan = kanamycin, MP = membrane protein, sfGFP = superfolder green fluorescent protein, UTR = untranslated region, TT = terminator region

**Supplementary Tables**

**Supplementary Table S1.** List of fold changes for NlpE and sfGFP expression (at OD_600_ = 0.3) measured for indirect IM stress analysis. Statistics and analysis of variance performed in biological triplicate (Col1, Col2 and Col3). Significantly different p-values (p < 0.05) are depicted in bold. Col = colony, mKate2 = red fluorescent protein, sfGFP = super folder green fluorescent protein, OD_600_ = optical density measured at 600 nm, IM = inner-membrane, IPTG = isopropyl β-D-thiogalactopyranoside

| **NlpE + P*_cpx_*_P(+5)_-mKate2** | | | | |
| --- | --- | --- | --- | --- |
| IPTG (mM) | Fold Change | | | |
|  | Col1 | Col2 | Col3 | Mean |
| 0 | 0.984 | 0.981 | 1.035 | 1.0 |
| 0.01 | 2.871 | 3.535 | 4.078 | 3.495 |
| 0.05 | 7.924 | 6.777 | 8.655 | 7.785 |
| ANOVA for fitted linear model | | | | |
| df | | F-value | | p-value |
| 7 | | 98.956 | | **0.022E-3** |
| **sfGFP + P*_cpx_*_P(+5)_-mKate2** | | | | |
| IPTG (mM) | Fold Change | | | |
|  | Col1 | Col2 | Col3 | Mean |
| 0 | 1.050 | 0.985 | 0.965 | 1.0 |
| 0.01 | 1.033 | 0.990 | 1.010 | 1.01 |
| 0.05 | 1.035 | 0.960 | 1.087 | 1.027 |
| ANOVA for fitted linear model | | | | |
| df | | F-value | | p-value |
| 7 | | 0.578 | | 0.472 |

**Supplementary Table S2.** Bacterial *Escherichia coli* strains that were used in this study. * Strain DE3 was kindly provided by G. Stephanopoulos of the Massachusetts Institute of Technology (MIT).

| **Trivial name** | **Genotype** | **Purpose** | **Source** |
| --- | --- | --- | --- |
| TOP10 | *E. coli* One Shot Top10 Electrocomp^TM^ | General cloning | Life Technologies |
| DH10B | *E. coli* DH10B (*ara*D139Δ(*ara,leu*)) | Expression strain | Thermofisher ElectroMAX^TM^ |
| DE3 | *E. coli* MG1656 DE3 Δ*end*AΔ*rec*A | Expression strain | MIT* |

**Supplementary Table S3.** List of DNA sequences used in this study to design the smart small RNA library. Bold nucleotides are different from the native *cpx*Q sRNA. sRNA = small RNA

| **sRNA** | **DNA sequence** |
| --- | --- |
| *cpx*Q | 5’-TTT TCC TTG CCA TAG ACA CCA TCC CTG TCT TCC CCC ACA TGC TGT GGG GGT TTT TTT T-3’ |
| *cpx*Qmut1 | 5’-**A**TT TCC TT**C** **TT**A TAG ACA CCA TCC CTG TCT TCC CCC ACA TGC TGT GGG GGT TTT TTT T-3’ |
| *cpx*Qmut2 | 5’-**A**TT TCC TT**C** **TT**A **AT**G ACA CCA TCC CTG TCT TCC CCC ACA TGC TGT GGG GGT TTT TTT T-3’ |
| *cpx*Qmut3 | 5’-**A**TT TCC TT**C** **TT**A **A**AG ACA CCA TCC CTG TCT TCC CCC ACA TGC TGT GGG GGT TTT TTT T-3’ |
| *cpx*Qmut4 | 5’-**A**TT TCC TT**C** **TT**A **A**AG **TT**A **AAC** TCC CTG TCT TCC CCC ACA TGC TGT GGG GGT TTT TTT T-3’ |
| *cpx*Qmut5 | 5’-**A**TT TCC TT**C TT**A TAG **TT**A **AAC AAA A**T**T** TCT TCC CCC ACA TGC TGT GGG GGT TTT TTT T-3’ |
| *cpx*Qmut6 | 5’-**A**TT TCC TT**C TT**A TAG **TT**A **AAC** **AAA A**T**T** **AT**T TCC CCC ACA TGC TGT GGG GGT TTT TTT T-3’ |
| *cpx*Qmut7 | 5’-**A**TT TCC TT**C TT**A TAG **TT**A **AAC** **AAA A**T**T** **AT**T TC**T** **AGA** ACA TGC TGT GGG GGT TTT TTT T-3’ |
| *cpx*Qmut8 | 5’-**A**TT TCC TT**C TT**A **A**AG **TT**A **AAC** **AAA A**T**T** **AT**T TC**T** **A**CC ACA TGC TGT GGG GGT TTT TTT T-3’ |
| *cpx*Qmut9 | 5’-**A**TT TCC TT**C TT**A **A**AG **TT**A **AAC** **AAA A**T**T** **AT**T TCC CCC ACA TGC TGT GGG GGT TTT TTT T-3’ |
| *cpx*Qmut non-specific | 5’-TTT **CCT** **CC**G CCA TAG ACA CCA TCC CTG TCT TCC CCC ACA TGC TGT GGG GGT TTT TTT T-3’ |

**Supplementary Table S4.** Two sample t-tests performed in this study. Tests are described using DFtotal for the total degrees of freedom. Only significant t-tests are depicted in bold. trunc = truncated

| **Test-details – sfGFP/mKate2** | **p-value** | **DFtotal** |
| --- | --- | --- |
| sfGFP(trunc)_sfGFP and P_BAD_-*cpx*Qmut1_sfGFP (0% L-arabinose) | 0.3 | 2 |
| sfGFP(trunc)_sfGFP and P_BAD_-*cpx*Qmut1_sfGFP (0.2% L-arabinose) | **1.14E-05** | 2 |
| sfGFP(trunc)_sfGFP and P_BAD_-*cpx*Qmut1_sfGFP (0.5% L-arabinose) | **4.15E-06** | 2 |
| sfGFP(trunc)_sfGFP and P_BAD_-*cpx*Qmut1_sfGFP (1% L-arabinose) | **8.71E-06** | 2 |
| **Test-details – sfGFP/OD_600_** | **p-value** | **DFtotal** |
| sfGFP(trunc)_sfGFP and P_BAD_-*cpx*Qmut1_sfGFP (0% L-arabinose) | **0.047** | 2 |
| sfGFP(trunc)_sfGFP and P_BAD_-*cpx*Qmut1_sfGFP (0.2% L-arabinose) | **7.50E-07** | 2 |
| sfGFP(trunc)_sfGFP and P_BAD_-*cpx*Qmut1_sfGFP (0.5% L-arabinose) | **4.87E-06** | 2 |
| sfGFP(trunc)_sfGFP and P_BAD_-*cpx*Qmut1_sfGFP (1% L-arabinose) | **2.19E-05** | 2 |
| **Test-details – mKate2** | **p-value** | **DFtotal** |
| sfGFP(trunc)_sfGFP and P_BAD_-*cpx*Qmut1_sfGFP (0% L-arabinose) | **0.012** | 2 |
| sfGFP(trunc)_sfGFP and P_BAD_-*cpx*Qmut1_sfGFP (0.2% L-arabinose) | **0.13E-03** | 2 |
| sfGFP(trunc)_sfGFP and P_BAD_-*cpx*Qmut1_sfGFP (0.5% L-arabinose) | **0.52E-03** | 2 |
| sfGFP(trunc)_sfGFP and P_BAD_-*cpx*Qmut1_sfGFP (1% L-arabinose) | **4.94E-05** | 2 |
| **Test-details – sfGFP** | **p-value** | **DFtotal** |
| sfGFP(trunc)_sfGFP and P_BAD_-*cpx*Qmut1_sfGFP (0% L-arabinose) | **0.045** | 2 |
| sfGFP(trunc)_sfGFP and P_BAD_-*cpx*Qmut1_sfGFP (0.2% L-arabinose) | **2.96E-06** | 2 |
| sfGFP(trunc)_sfGFP and P_BAD_-*cpx*Qmut1_sfGFP (0.5% L-arabinose) | **7.98E-07** | 2 |
| sfGFP(trunc)_sfGFP and P_BAD_-*cpx*Qmut1_sfGFP (1% L-arabinose) | **1.46E-06** | 2 |
| **Test-details – µ_max_** | **p-value** | **DFtotal** |
| sfGFP(trunc)_sfGFP and P_BAD_-*cpx*Qmut1_sfGFP (0% L-arabinose) | **0.041** | 2 |
| sfGFP(trunc)_sfGFP and P_BAD_-*cpx*Qmut1_sfGFP (0.2% L-arabinose) | **0.02** | 2 |
| sfGFP(trunc)_sfGFP and P_BAD_-*cpx*Qmut1_sfGFP (0.5% L-arabinose) | 0.75 | 2 |
| sfGFP(trunc)_sfGFP and P_BAD_-*cpx*Qmut1_sfGFP (1% L-arabinose) | 0.24 | 2 |
| **Test-details – sfGFP/OD_600_** | **p-value** | **DFtotal** |
| SohB(TMD)-sfGFP and *cpx*Qmut non-specific (0.2 mM IPTG) | 0.052 | 2 |
| GarP-sfGFP and *cpx*Qmut non-specific (0.2 mM IPTG) | 0.058 | 2 |
| YidC-sfGFP and *cpx*Qmut non-specific (0.2 mM IPTG) | 0.30 | 2 |

**Supplementary Table S5.** Statistical one-way ANOVA performed in this study. Tests are described using DFtotal for the total degrees of freedom. Only significant post hoc tests are depicted italic directly under the corresponding ANOVA. Significantly different p-values (p < 0.05) are depicted in bold. µ_max_ = maximum specific growth rate (h^−1^), sfGFP = superfolder green fluorescent protein, SohB = inner-membrane protein in *Escherichia coli*, TMD = transmembrane domain

| **Test-details** | **p-value** | **DFtotal** |
| --- | --- | --- |
| **ANOVA µ_max_ design evaluation (0.1 mM IPTG)** | 0.087 | 14 |
| *Tuckey HSD:* *SohB(TMD)-sfGFP (Ref) and D1* | 0.628 | 10 |
| *Tuckey HSD:* *SohB(TMD)-sfGFP (Ref) and D2* | 0.357 | 10 |
| *Tuckey HSD:* *SohB(TMD)-sfGFP (Ref) and D3* | 0.328 | 10 |
| *Tuckey HSD:* *SohB(TMD)-sfGFP (Ref) and D4* | 0.053 | 10 |
| **ANOVA µ_max_ design evaluation (0.2 mM IPTG)** | **0.008** | 14 |
| *Tuckey HSD:* *SohB(TMD)-sfGFP (Ref) and D1* | 0.9 | 10 |
| *Tuckey HSD:* *SohB(TMD)-sfGFP (Ref) and D2* | 0.9 | 10 |
| *Tuckey HSD:* *SohB(TMD)-sfGFP (Ref) and D3* | 0.9 | 10 |
| *Tuckey HSD:* *SohB(TMD)-sfGFP (Ref) and D4* | **0.018** | 10 |
| **ANOVA Fold Change sfGFP design evaluation (0.1 mM IPTG)** | 0.453 | 14 |
| *Tuckey HSD:* *SohB(TMD)-sfGFP (Ref) and D1* | 0.9 | 10 |
| *Tuckey HSD:* *SohB(TMD)-sfGFP (Ref) and D2* | 0.757 | 10 |
| *Tuckey HSD:* *SohB(TMD)-sfGFP (Ref) and D3* | 0.674 | 10 |
| *Tuckey HSD:* *SohB(TMD)-sfGFP (Ref) and D4* | 0.367 | 10 |
| **ANOVA Fold Change sfGFP design evaluation (0.2 mM IPTG)** | **0.00035** | 14 |
| *Tuckey HSD:* *SohB(TMD)-sfGFP (Ref) and D1* | 0.9 | 10 |
| *Tuckey HSD:* *SohB(TMD)-sfGFP (Ref) and D2* | 0.9 | 10 |
| *Tuckey HSD:* *SohB(TMD)-sfGFP (Ref) and D3* | 0.9 | 10 |
| *Tuckey HSD:* *SohB(TMD)-sfGFP (Ref) and D4* | **0.001** | 10 |
| **ANOVA sfGFP/OD_600_ design evaluation (0.1 mM IPTG)** | 0.05 | 14 |
| *Tuckey HSD:* *SohB(TMD)-sfGFP (Ref) and D1* | 0.287 | 10 |
| *Tuckey HSD:* *SohB(TMD)-sfGFP (Ref) and D2* | 0.244 | 10 |
| *Tuckey HSD:* *SohB(TMD)-sfGFP (Ref) and D3* | 0.9 | 10 |
| *Tuckey HSD:* *SohB(TMD)-sfGFP (Ref) and D4* | 0.063 | 10 |
| **ANOVA sfGFP/OD_600_ design evaluation (0.2 mM IPTG)** | **0.024** | 14 |
| *Tuckey HSD:* *SohB(TMD)-sfGFP (Ref) and D1* | 0.9 | 10 |
| *Tuckey HSD:* *SohB(TMD)-sfGFP (Ref) and D2* | 0.867 | 10 |
| *Tuckey HSD:* *SohB(TMD)-sfGFP (Ref) and D3* | 0.9 | 10 |
| *Tuckey HSD:* *SohB(TMD)-sfGFP (Ref) and D4* | **0.025** | 10 |
| **ANOVA µ_max_ smart sRNA library** | **2.251E-08** | 35 |
| *Tuckey HSD:* *SohB(TMD)-sfGFP and cpxQ* | 0.9 | 24 |
| *Tuckey HSD:* *SohB(TMD)-sfGFP and cpxQmut1* | **0.009** | 24 |
| *Tuckey HSD:* *SohB(TMD)-sfGFP and cpxQmut2* | 0.9 | 24 |
| *Tuckey HSD:* *SohB(TMD)-sfGFP and cpxQmut3* | **0.001** | 24 |
| *Tuckey HSD:* *SohB(TMD)-sfGFP and cpxQmut4* | 0.9 | 24 |
| *Tuckey HSD:* *SohB(TMD)-sfGFP and cpxQmut5* | **0.048** | 24 |
| *Tuckey HSD:* *SohB(TMD)-sfGFP and cpxQmut6* | **0.048** | 24 |
| *Tuckey HSD:* *SohB(TMD)-sfGFP and cpxQmut7* | 0.516 | 24 |
| *Tuckey HSD:* *SohB(TMD)-sfGFP and cpxQmut8* | 0.9 | 24 |
| *Tuckey HSD:* *SohB(TMD)-sfGFP and cpxQmut9* | **0.002** | 24 |
| *Tuckey HSD:* *SohB(TMD)-sfGFP and sfGFP* | **0.001** | 24 |
| **ANOVA Fold Change sfGFP smart sRNA library** | **1.124E-07** | 32 |
| *Tuckey HSD:* *SohB(TMD)-sfGFP and cpxQ* | 0.9 | 22 |
| *Tuckey HSD:* *SohB(TMD)-sfGFP and cpxQmut1* | **0.001** | 22 |
| *Tuckey HSD:* *SohB(TMD)-sfGFP and cpxQmut2* | 0.117 | 22 |
| *Tuckey HSD:* *SohB(TMD)-sfGFP and cpxQmut3* | **0.001** | 22 |
| *Tuckey HSD:* *SohB(TMD)-sfGFP and cpxQmut4* | 0.9 | 22 |
| *Tuckey HSD:* *SohB(TMD)-sfGFP and cpxQmut5* | 0.092 | 22 |
| *Tuckey HSD:* *SohB(TMD)-sfGFP and cpxQmut6* | **0.016** | 22 |
| *Tuckey HSD:* *SohB(TMD)-sfGFP and cpxQmut7* | 0.650 | 22 |
| *Tuckey HSD:* *SohB(TMD)-sfGFP and cpxQmut8* | 0.9 | 22 |
| *Tuckey HSD:* *SohB(TMD)-sfGFP and cpxQmut9* | **0.013** | 22 |
| **ANOVA sfGFP/OD_600_ smart sRNA library** | **3.170E-05** | 32 |
| *Tuckey HSD:* *SohB(TMD)-sfGFP and cpxQ* | 0.9 | 22 |
| *Tuckey HSD:* *SohB(TMD)-sfGFP and cpxQmut1* | **0.004** | 22 |
| *Tuckey HSD:* *SohB(TMD)-sfGFP and cpxQmut2* | **0.042** | 22 |
| *Tuckey HSD:* *SohB(TMD)-sfGFP and cpxQmut3* | **0.001** | 22 |
| *Tuckey HSD:* *SohB(TMD)-sfGFP and cpxQmut4* | 0.194 | 22 |
| *Tuckey HSD:* *SohB(TMD)-sfGFP and cpxQmut5* | **0.022** | 22 |
| *Tuckey HSD:* *SohB(TMD)-sfGFP and cpxQmut6* | **0.002** | 22 |
| *Tuckey HSD:* *SohB(TMD)-sfGFP and cpxQmut7* | 0.370 | 22 |
| *Tuckey HSD:* *SohB(TMD)-sfGFP and cpxQmut8* | 0.702 | 22 |
| *Tuckey HSD:* *SohB(TMD)-sfGFP and cpxQmut9* | **0.001** | 22 |
| **ANOVA µ_max_ MP overexpression (SohB(TMD)-sfGFP)** | **5.58E-05** | 8 |
| *Tuckey HSD:* *SohB(TMD)-sfGFP and cpxQmut1* | **0.001** | 6 |
| *Tuckey HSD:* *SohB(TMD)-sfGFP and cpxQmut3* | **0.001** | 6 |
| **ANOVA µ_max_ MP overexpression (YidC-sfGFP)** | **3.47E-05** | 8 |
| *Tuckey HSD:* *YidC-sfGFP and cpxQmut1* | **0.001** | 6 |
| *Tuckey HSD:* *YidC-sfGFP and cpxQmut3* | **0.001** | 6 |
| **ANOVA µ_max_ MP overexpression (GarP-sfGFP)** | **1.26E-08** | 8 |
| *Tuckey HSD: GarP-sfGFP and cpxQmut1* | **0.001** | 6 |
| *Tuckey HSD: GarP-sfGFP and cpxQmut3* | **0.001** | 6 |
| **ANOVA Fold Change sfGFP MP overexpression (SohB(TMD)-sfGFP)** | **8.10E-07** | 8 |
| *Tuckey HSD:* *SohB(TMD)-sfGFP and cpxQmut1* | **0.001** | 6 |
| *Tuckey HSD:* *SohB(TMD)-sfGFP and cpxQmut3* | **0.001** | 6 |
| **ANOVA Fold Change sfGFP MP overexpression (YidC-sfGFP)** | **0.957E-03** | 8 |
| *Tuckey HSD:* *YidC-sfGFP and cpxQmut1* | **0.016** | 6 |
| *Tuckey HSD:* *YidC-sfGFP and cpxQmut3* | **0.001** | 6 |
| **ANOVA Fold Change sfGFP MP overexpression (GarP-sfGFP)** | **0.153E-03** | 8 |
| *Tuckey HSD:* *GarP-sfGFP and cpxQmut1* | **0.010** | 6 |
| *Tuckey HSD:* *GarP-sfGFP and cpxQmut3* | **0.001** | 6 |
| **ANOVA sfGFP/OD_600_ MP overexpression (SohB(TMD)-sfGFP)** | **9.570E-06** | 8 |
| *Tuckey HSD:* *SohB(TMD)-sfGFP and cpxQmut1* | **0.001** | 6 |
| *Tuckey HSD:* *SohB(TMD)-sfGFP and cpxQmut3* | **0.001** | 6 |
| **ANOVA sfGFP/OD_600_ MP overexpression (YidC-sfGFP)** | **4.640E-08** | 8 |
| *Tuckey HSD:* *YidC-sfGFP and cpxQmut1* | **0.001** | 6 |
| *Tuckey HSD:* *YidC-sfGFP and cpxQmut3* | **0.001** | 6 |
| **ANOVA sfGFP/OD_600_ MP overexpression (GarP-sfGFP)** | **6.895E-04** | 8 |
| *Tuckey HSD:* *GarP-sfGFP and cpxQmut1* | **0.001** | 6 |
| *Tuckey HSD:* *GarP-sfGFP and cpxQmut3* | **0.001** | 6 |
| **ANOVA µ_max_ MP overexpression (SohB(TMD)-sfGFP)** | **2.12E-08** | 14 |
| *Tuckey HSD:* *SohB(TMD)-sfGFP and cpxQmut non-specific* | 0.11 | 11 |
| *Tuckey HSD:* *SohB(TMD)-sfGFP and cpxQmut1* | **0.001** | 11 |
| *Tuckey HSD:* *SohB(TMD)-sfGFP and cpxQmut3* | **0.001** | 11 |
| **ANOVA µ_max_ MP overexpression (GarP-sfGFP)** | **3.78E-07** | 14 |
| *Tuckey HSD:* *GarP-sfGFP and cpxQmut non-specific* | 0.20 | 11 |
| *Tuckey HSD:* *GarP-sfGFP and cpxQmut1* | **0.001** | 11 |
| *Tuckey HSD:* *GarP-sfGFP and cpxQmut3* | **0.001** | 11 |
| **ANOVA µ_max_ MP overexpression (YidC-sfGFP)** | **5.35E-08** | 14 |
| *Tuckey HSD:* *YidC-sfGFP and cpxQmut non-specific* | 0.9 | 11 |
| *Tuckey HSD:* *YidC-sfGFP and cpxQmut1* | **0.001** | 11 |
| *Tuckey HSD:* *YidC-sfGFP and cpxQmut3* | **0.001** | 11 |

**Supplementary Table S6.** Plasmids that were used and constructed throughout this study. Genetic determinants driving genes are represented as Promoter(5’UTR). TT stands for transcription terminator, TMD = transmembrane domain, Ct = C-terminus, Nt = N-terminus

| **Indirect IM stress analysis** | Plasmid details | |
| --- | --- | --- |
| sfGFP (negative control) | pET22B_AmpR_P_T7_(T7)_*sfgfp*_TT7 | |
| NlpE (positive control) | pET22B_AmpR_P_T7_(T7)_*nlp*E_TT7 | |
| P*_cpx_*_P(+5)_-mKate2 | pSC101_KanR_P*_cpx_*_P(+5)_(proB(5’UTR))_*mKate2*_BioFabTT | |
| **small RNA engineering** |  | |
| pCP(1-2) | pCP(2-3) | pEX-BR322: P*_pro_*_B_-*mKate2*-BioFabTT |
| araC-P_BAD_-cpxQmut1 | P_14_(T7)-*sfgfp*-TT7(M13) | (1-3) |
| araC-P_BAD_-cpxQmut2 |  |  |
| araC-P_BAD_-cpxQmut3 |  |  |
| sfGFP(trunc) |  |  |
| Final constructs –  GG assembly | Plasmid details | |
| P_BAD_-cpxQmut1_sfGFP | pBR322_KanR_*ara*C_P_BAD_-*cpx*Qmut1_P_14_(T7)_*sfgfp*_TT7_P*_pro_*_B__*mKate2*_BioFabTT | |
| P_BAD_-cpxQmut2_sfGFP | pBR322_KanR_*ara*C_P_BAD_-*cpx*Qmut2_P_14_(T7)_*sfgfp*_TT7_ P*_pro_*_B__*mKate2*_BioFabTT | |
| P_BAD_-cpxQmut3_sfGFP | pBR322_KanR_*ara*C_P_BAD_-*cpx*Qmut3_P_14_(T7)_*sfgfp*_TT7_ P*_pro_*_B__*mKate2*_BioFabTT | |
| sfGFP(trunc)_sfGFP | pBR322_KanR_*sfgfp(trunc)*_P_14_(T7)_*sfgfp*_TT7_P*_pro_*_B__*mKate2*_BioFabTT | |
| **Evaluation of TMDs** | Plasmid details | |
| SohB(TMD)-sfGFP | pET22B_AmpR_P_T7_(T7)_*soh*B(TMD)_*sfgfp*_TT7 | |
| YhcB(TMD)-sfGFP | pET22B_AmpR_P_T7_(T7)_*yhc*B(TMD)_*sfgfp*_TT7 | |
| 17αCYP(TMD)-sfGFP | pET22B_AmpR_P_T7_(T7)_*17αCYP*(TMD)_*sfgfp*_TT7 | |
| PelB(SP)-sfGFP | pET22B_AmpR_P_T7_(T7)_*pel*B(SP)_*sfgfp*_TT7 | |
| **Design evaluation** | Plasmid details | |
| SohB(TMD)-sfGFP (Ref) | pET22B_AmpR_P_T7_(T7)_*soh*B(TMD)_*sfgfp*_TT7 | |
| P*_cpx_*_P_-cpxP (pD1) | pSC101_KanR_P*_cpx_*_P_(5’UTR cpxP)-*cpx*P-*cpx*Qmut1 | |
| P*_cpx_*_P_-cpxQmut1 (pD2) | pSC101_KanR_P*_cpx_*_P_-*cpx*Qmut1 | |
| P*_cpx_*_P(+5)_-cpxQmut1 (pD3) | pSC101_KanR_P*_cpx_*_P(+5)_-*cpx*Qmut1 | |
| SohB(TMD)-sfGFP-cpxQmut1 (D4) | pET22B_ AmpR_P_T7_(T7)_*soh*B(TMD)_*sfgfp*_TT7-P*_cpx_*_P(+5)_-*cpx*Qmut1 | |
| **Smart small RNA library** | Plasmid details | |
| SohB(TMD)-sfGFP (Ref) | pET22B_ AmpR_P_T7_(T7)_*soh*B(TMD)_*sfgfp*_TT7 | |
| *cpx*Q | pET22B_ AmpR_P_T7_(T7)_*soh*B(TMD)_*sfgfp*_TT7_P*_cpx_*_P(+5)_-*cpx*Q | |
| *cpx*Qmut1 | pET22B_ AmpR_P_T7_(T7)_*soh*B(TMD)_*sfgfp*_TT7_P*_cpx_*_P(+5)_-*cpx*Qmut1 | |
| *cpx*Qmut2 | pET22B_ AmpR_P_T7_(T7)_*soh*B(TMD)_*sfgfp*_TT7_P*_cpx_*_P(+5)_-*cpx*Qmut2 | |
| *cpx*Qmut3 | pET22B_ AmpR_P_T7_(T7)_*soh*B(TMD)_*sfgfp*_TT7_P*_cpx_*_P(+5)_-*cpx*Qmut3 | |
| *cpx*Qmut4 | pET22B_ AmpR_P_T7_(T7)_*soh*B(TMD)_*sfgfp*_TT7_P*_cpx_*_P(+5)_-*cpx*Qmut4 | |
| *cpx*Qmut5 | pET22B_ AmpR_P_T7_(T7)_*soh*B(TMD)_*sfgfp*_TT7_P*_cpx_*_P(+5)_-*cpx*Qmut5 | |
| *cpx*Qmut6 | pET22B_ AmpR_P_T7_(T7)_*soh*B(TMD)_*sfgfp*_TT7_P*_cpx_*_P(+5)_-*cpx*Qmut6 | |
| *cpx*Qmut7 | pET22B_ AmpR_P_T7_(T7)_*soh*B(TMD)_*sfgfp*_TT7_P*_cpx_*_P(+5)_-*cpx*Qmut7 | |
| *cpx*Qmut8 | pET22B_ AmpR_P_T7_(T7)_*soh*B(TMD)_*sfgfp*_TT7_P*_cpx_*_P(+5)_-*cpx*Qmut8 | |
| *cpx*Qmut9 | pET22B_ AmpR_P_T7_(T7)_*soh*B(TMD)_*sfgfp*_TT7_P*_cpx_*_P(+5)_-*cpx*Qmut9 | |
| **MP overproduction** | Plasmid details | |
| sfGFP | pET22B_AmpR_P_T7_(T7)_*sfgfp*_TT7 | |
| SohB(TMD)-sfGFP | pET22B_ AmpR_P_T7_(T7)_*soh*B(TMD)_*sfgfp*_TT7 | |
| SohB(TMD)-sfGFP-*cpx*Qmut1/3 | pET22B_ AmpR_P_T7_(T7)_*soh*B(TMD)_*sfgfp*_TT7_P*_cpx_*_P(+5)_-*cpx*Qmut1/3 | |
| YidC-sfGFP | pET22B_ AmpR_P_T7_(T7)_*yid*C_linker(GC)_*sfgfp*_TT7 | |
| YidC-sfGFP-*cpx*Qmut1/3 | pET22B_ AmpR_P_T7_(T7)_*yid*C_linker(GC)_*sfgfp*_TT7_P*_cpx_*_P(+5)_-*cpx*Qmut1/3 | |
| GarP-sfGFP | pET22B_ AmpR_P_T7_(T7)_*gar*P_linker(GC)_*sfgfp*_TT7 | |
| GarP-sfGFP-*cpx*Qmut1/3 | pET22B_ AmpR_P_T7_(T7)_*gar*P_linker(GC)_*sfgfp*_TT7_P*_cpx_*_P(+5)_-*cpx*Qmut1/3 | |
| SohB(TMD)-sfGFP-*cpx*Qmut non-specific | pET22B_ AmpR_P_T7_(T7)_ *soh*B(TMD)_*sfgfp*_TT7_P*_cpx_*_P(+5)_-*cpx*Qmut non-specific | |
| YidC-sfGFP-*cpx*Qmut non-specific | pET22B_ AmpR_P_T7_(T7)_*yid*C_linker(GC)_*sfgfp*_TT7_P*_cpx_*_P(+5)_-*cpx*Qmut non-specific | |
| GarP-sfGFP-*cpx*Qmut non-specific | pET22B_ AmpR_P_T7_(T7)_*gar*P_linker(GC)_*sfgfp*_TT7_P*_cpx_*_P(+5)_-*cpx*Qmut non-specific | |

**Supplementary Table S7.** List of proteins used in this study for membrane localisation and membrane targeting. IM = inner-membrane, MP = membrane protein, TM = transmembrane domain, TMD = transmembrane domain

| **Abbreviation** | **Organism of origin** | **Function** | **Reference** |
| --- | --- | --- | --- |
| sfGFP | *Aequoria victoria* | Super folder green fluorescent protein | (4) |
| mKate2 | *Entacmaea quadricolor* | Red fluorescent protein | (5) |
| SohB(TMD) | *Escherichia coli* | IM protein | Genbank ID 945858 |
| YhcB(TMD) | *Escherichia coli* | Putative subunit of membrane-associated protein complex | Genbank ID 947815 |
| PelB | *Erwinia carotovora* | Signal peptide | (6) |
| 17αCYP | Bovine | Cytochrome P450 | (7) |
| YidC | *Escherichia coli* | MP insertase | Genbank ID 948214 |
| GarP | *Escherichia coli* | TM transporter | Genbank ID 947642 |
| NlpE | *Escherichia coli* | IM lipoprotein | Genbank ID 946782 |

**Supplementary Table S8.** List of fold changes for NlpE, sfGFP, SohB(TMD)-sfGFP, YidC-sfGFP and GarP-sfGFP expression (at t = 20h) measured for indirect IM stress analysis. Statistics and analysis of variance performed in biological triplicate (Col1, Col2 and Col3). Significantly different p-values (p < 0.05) are depicted in bold. Col = colony, mKate2 = red fluorescent protein, sfGFP = super folder green fluorescent protein, IM = inner-membrane, IPTG = isopropyl β-D-thiogalactopyranoside, YidC = membrane protein insertase in *E. coli*, GarP = TM transporter in *E. coli,* SohB(TMD)= transmembrane domain of inner-membrane protein SohB from *E. coli*

| **NlpE + P*_cpx_*_P(+5)_-mKate2** | | | | |
| --- | --- | --- | --- | --- |
| IPTG (mM) | Fold Change | | | |
|  | Col1 | Col2 | Col3 | Mean |
| 0 | 1.042 | 1.004 | 0.954 | 1.0 |
| 0.01 | 5.025 | 5.735 | 4.978 | 5.246 |
| 0.05 | 25.849 | 39.047 | 31.141 | 32.013 |
| 0.1 | 21.443 | 19.980 | 13.319 | 18.245 |
| ANOVA for fitted linear model | | | | |
| df | | F-value | | p-value |
| 10 | | 6.116 | | **0.033** |
| **sfGFP + P*_cpx_*_P(+5)_-mKate2** | | | | |
| IPTG (mM) | Fold Change | | | |
|  | Col1 | Col2 | Col3 | Mean |
| 0 | 1.037 | 0.971 | 0.992 | 1.0 |
| 0.01 | 1.101 | 0.948 | 0.934 | 0.995 |
| 0.05 | 1.259 | 1.049 | 1.065 | 1.124 |
| 0.1 | 1.635 | 1.407 | 1.548 | 1.530 |
| ANOVA for fitted linear model | | | | |
| df | | F-value | | p-value |
| 10 | | 47.206 | | **0.043E-3** |
| **SohB(TMD)-sfGFP + P*_cpx_*_P(+5)_-mKate2** | | | | |
| IPTG (mM) | Fold Change | | | |
|  | Col1 | Col2 | Col3 | Mean |
| 0 | 1.015 | 0.982 | 1.003 | 1.0 |
| 0.01 | 0.795 | 0.790 | 0.740 | 0.775 |
| 0.05 | 4.896 | 4.919 | 4.718 | 4.844 |
| 0.1 | 5.669 | 6.829 | 5.899 | 6.132 |
| ANOVA for fitted linear model | | | | |
| df | | F-value | | p-value |
| 10 | | 92.714 | | **0.002E-3** |
| **YidC-sfGFP + P*_cpx_*_P(+5)_-mKate2** | | | | |
| IPTG (mM) | Fold Change | | | |
|  | Col1 | Col2 | Col3 | Mean |
| 0 | 1.016 | 0.959 | 1.024 | 1.0 |
| 0.01 | 5.026 | 4.725 | 4.318 | 4.690 |
| 0.05 | 30.141 | 28.456 | 23.749 | 27.449 |
| 0.1 | 23.703 | 18.919 | 25.518 | 22.713 |
| ANOVA for fitted linear model | | | | |
| df | | F-value | | p-value |
| 10 | | 18.457 | | **0.002** |
| **GarP-sfGFP + P*_cpx_*_P(+5)_-mKate2** | | | | |
| IPTG (mM) | Fold Change | | | |
|  | Col1 | Col2 | Col3 | Mean |
| 0 | 1.071 | 0.930 | 0.999 | 1.0 |
| 0.01 | 1.487 | 1.350 | 4.146 | 2.328 |
| 0.05 | 3.232 | 2.536 | 2.918 | 2.895 |
| 0.1 | 3.886 | 3.573 | 3.278 | 3.579 |
| ANOVA for fitted linear model | | | | |
| df | | F-value | | p-value |
| 10 | | 11.550 | | **0.007** |

**Supplementary Table S9.** Overview of the different plasmid backbones used in this study and their assigned function. Amp = ampicillin, Chlor = chloramphenicol, IM = inner-membrane, Kan = kanamycin

| **Plasmid** | **Copy number** | **Antibiotic** | **Reference** |
| --- | --- | --- | --- |
| **Production plasmids**  pET22B | ~ 15-20 | Amp | Invitrogen |
| **Expression plasmids (pEX)**  pBR322 | ~15-20 | Kan/Chlor | (2) |
| **Carrier plasmids**  pBR322  pUC | ~15-20  ~500-700 | Amp  Kan | (2)  (2) |
| **Detection IM stress +**  **Design evaluation**  pSC101 | ~5 | Kan | (3) |

**Supplementary Table S10.** List of DNA sequences used in this study (coding sequences, promoter, 5’UTRs and terminator sequences). For coding sequences, start and stop codons are indicated with lower-case letters. sf stands for super folder, UTR = untranslated region

| **Gene** | **Coding sequence** |
| --- | --- |
| *nlp*E | 5’-atg GTG AAA AAA GCG ATA GTG ACA GCG ATG GCT GTA ATC AGC CTC TTT ACT CTG ATG GGA TGT AAT AATCGG GCC GAA GTC GAT ACG CTT TCT CCG GCG CAG GCT GCC GAA CTG AAA CCG ATG CCG CAA AGT TGG CGCGGC GTG CTG CCG TGT GCC GAT TGC GAA GGA ATC GAA ACC TCT CTG TTC CTC GAA AAA GAC GGA ACA TGGGTG ATG AAT GAG CGT TAT CTC GGT GCT CGT GAA GAA CCT TCC TCC TTC GCT TCC TAC GGT ACA TGG GCGCGA ACC GCT GAC AAG CTG GTA TTA ACC GAC AGC AAA GGT GAA AAG TCA TAT TAT CGG GCG AAA GGC GATGCG CTG GAG ATG CTC GAT CGT GAA GGC AAT CCG ATT GAA TCG CAG TTC AAC TAT ACG CTG GAA GCG GCACAA TCC AGT TTA CCT ATG ACG CCG ATG ACC CTG CGG GGC ATG TAT TTT TAT ATG GCT GAT GCG GCG ACCTTC ACT GAT TGC GCG ACC GGA AAA CGT TTC ATG GTA GCG AAT AAC GCA GAG CTG GAG CGT AGC TAC CTGGCT GCG CGC GGT CAC AGT GAA AAA CCG GTG TTA CTG TCA GTA GAA GGT CAC TTT ACG CTT GAG GGT AATCCG GAT ACC GGT GCG CCG ACT AAA GTA TTG GCA CCC GAT ACG GCA GGG AAA TTT TAC CCC AAC CAG GATTGC AGT AGT TTG GGG CAG taa-3 |
| *mKate2* | 5’-atg GTT AGC GAG CTG ATC AAA GAA AAC ATG CAC ATG AAA CTG TAT ATG GAA GGC ACC GTG AAT AAC CACCAC TTT AAA TGT ACC AGC GAA GGT GAA GGT AAA CCG TAT GAA GGC ACC CAG ACC ATG CGT ATT AAA GCAGTT GAA GGT GGT CCG CTG CCG TTT GCA TTT GAT ATT CTG GCA ACC AGC TTT ATG TAT GGC AGC AAA ACCTTT ATT AAC CAT ACC CAG GGT ATC CCG GAT TTT TTC AAA CAG AGC TTT CCG GAA GGT TTT ACC TGG GAACGT GTT ACC ACC TAT GAA GAT GGT GGT GTT CTG ACC GCA ACC CAG GAT ACC AGT CTG CAG GAT GGT TGTCTG ATT TAT AAT GTG AAA ATT CGC GGT GTG AAC TTT CCG AGC AAT GGT CCG GTT ATG CAG AAA AAA ACCCTG GGT TGG GAA GCA AGC ACC GAA ACC CTG TAT CCG GCA GAT GGT GGT CTG GAA GGT CGT GCA GAT ATGGCA CTG AAA CTG GTT GGT GGT GGT CAT CTG ATT TGC AAT CTG AAA ACC ACC TAT CGT AGC AAA AAA CCGGCA AAA AAT CTG AAA ATG CCT GGC GTG TAT TAT GTT GAT CGT CGT CTG GAA CGT ATT AAA GAG GCA GATAAA GAA ACC TAT GTG GAA CAG CAT GAA GTT GCA GTT GCA CGT TAT TGT GAT CTG CCG AGC AAA CTG GGTCAC CGC tga-3’ |
| *sfgfp* | 5’-atg GGC AAG GGC GAA GAG CTT TTT ACC GGT GTT GTG CCG ATT TTA GTA GAA CTG GAC GGA GAC GTG AACGGT CAT AAG TTC TCT GTT CGT GGC GAA GGA GAG GGA GAT GCC ACC AAT GGT AAG CTG ACC CTG AAG TTCATC TGT ACC ACC GGT AAG CTG CCC GTG CCT TGG CCG ACG CTG GTC ACA ACG TTG ACG TAT GGC GTC CAATGC TTT TCA CGC TAT CCA GAT CAC ATG AAA CGC CAC GAC TTT TTT AAA AGC GCA ATG CCT GAA GGT TATGTG CAG GAA CGG ACT ATT AGC TTC AAA GAC GAT GGG ACG TAT AAG ACC CGC GCG GAA GTG AAA TTT GAAGGC GAT ACC TTA GTT AAC CGC ATT GAA TTA AAA GGT ATC GAT TTC AAA GAG GAT GGG AAT ATC CTG GGGCAC AAA TTG GAA TAC AAC TTT AAT TCG CAC AAC GTA TAC ATT ACA GCG GAT AAA CAG AAA AAT GGC ATCAAA GCC AAC TTT AAA ATC CGT CAT AAC GTA GAG GAC GGT TCC GTG CAG CTG GCT GAT CAT TAC CAG CAGAAT ACT CCG ATT GGC GAT GGC CCC GTT CTG CTC CCG GAT AAT CAT TAC CTG TCT ACA CAA AGC GTT CTTAGT AAA GAC CCA AAC GAG AAG CGT GAC CAT ATG GTC CTG TTG GAA TTC GTC ACG GCA GCG GGG ATT ACTCAT GGC ATG GAT GAA CTC TAT AAG taa-3’ |
| *lac*I | 5’-gtg AAA CCA GTA ACG TTA TAC GAT GTC GCA GAG TAT GCC GGT GTC TCT TAT CAG ACC GTT TCC CGC GTGGTG AAC CAG GCC AGC CAC GTT TCT GCG AAA ACG CGG GAA AAA GTG GAA GCG GCG ATG GCG GAG CTG AATTAC ATT CCC AAC CGC GTG GCA CAA CAA CTG GCG GGC AAA CAG TCG TTG CTG ATT GGC GTT GCC ACC TCCAGT CTG GCC CTG CAC GCG CCG TCG CAA ATT GTC GCG GCG ATT AAA TCT CGC GCC GAT CAA CTG GGT GCCAGC GTG GTG GTG TCG ATG GTA GAA CGA AGC GGC GTC GAA GCC TGT AAA GCG GCG GTG CAC AAT CTT CTCGCG CAA CGC GTC AGT GGG CTG ATC ATT AAC TAT CCG CTG GAT GAC CAG GAT GCC ATT GCT GTG GAA GCTGCC TGC ACT AAT GTT CCG GCG TTA TTT CTT GAT GTC TCT GAC CAG ACA CCC ATC AAC AGT ATT ATT TTCTCC CAT GAA GAC GGT ACG CGA CTG GGC GTG GAG CAT CTG GTC GCA TTG GGT CAC CAG CAA ATC GCG CTGTTA GCG GGC CCA TTA AGT TCT GTC TCG GCG CGT CTG CGT CTG GCT GGC TGG CAT AAA TAT CTC ACT CGCAAT CAA ATT CAG CCG ATA GCG GAA CGG GAA GGC GAC TGG AGT GCC ATG TCC GGT TTT CAA CAA ACC ATGCAA ATG CTG AAT GAG GGC ATC GTT CCC ACT GCG ATG CTG GTT GCC AAC GAT CAG ATG GCG CTG GGC GCAATG CGC GCC ATT ACC GAG TCC GGG CTG CGC GTT GGT GCG GAT ATC TCG GTA GTG GGA TAC GAC GAT ACCGAA GAC AGC TCA TGT TAT ATC CCG CCG TTA ACC ACC ATC AAA CAG GAT TTT CGC CTG CTG GGG CAA ACCAGC GTG GAC CGC TTG CTG CAA CTC TCT CAG GGC CAG GCG GTG AAG GGC AAT CAG CTG TTG CCC GTC TCACTG GTG AAA AGA AAA ACC ACC CTG GCG CCC AAT ACG CAA ACC GCC TCT CCC CGC GCG TTG GCC GAT TCATTA ATG CAG CTG GCA CGA CAG GTT TCC CGA CTG GAA AGC GGG CAG tga-3’ |
| *ara*C | 5’-atg GCT GAA GCG CAA AAT GAT CCC CTG CTG CCG GGA TAC TCG TTT AAT GCC CAT CTG GTG GCG GGT TTAACG CCG ATT GAG GCC AAC GGT TAT CTC GAT TTT TTT ATC GAC CGA CCG CTG GGA ATG AAA GGT TAT ATTCTC AAT CTC ACC ATT CGC GGT CAG GGG GTG GTG AAA AAT CAG GGA CGA GAA TTT GTT TGC CGA CCG GGTGAT ATT TTG CTG TTC CCG CCA GGA GAG ATT CAT CAC TAC GGT CGT CAT CCG GAG GCT CGC GAA TGG TATCAC CAG TGG GTT TAC TTT CGT CCG CGC GCC TAC TGG CAT GAA TGG CTT AAC TGG CCG TCA ATA TTT GCCAAT ACG GGG TTC TTT CGC CCG GAT GAA GCG CAC CAG CCG CAT TTC AGC GAC CTG TTT GGG CAA ATC ATTAAC GCC GGG CAA GGG GAA GGG CGC TAT TCG GAG CTG CTG GCG ATA AAT CTG CTT GAG CAA TTG TTA CTGCGG CGC ATG GAA GCG ATT AAC GAG TCG CTC CAT CCA CCG ATG GAT AAT CGG GTA CGC GAG GCT TGT CAGTAC ATC AGC GAT CAC CTG GCA GAC AGC AAT TTT GAT ATC GCC AGC GTC GCA CAG CAT GTT TGC TTG TCGCCG TCG CGT CTG TCA CAT CTT TTC CGC CAG CAG TTA GGG ATT AGC GTC TTA AGC TGG CGC GAG GAC CAACGT ATC AGC CAG GCG AAG CTG CTT TTG AGC ACC ACC CGG ATG CCT ATC GCC ACC GTC GGT CGC AAT GTTGGT TTT GAC GAT CAA CTC TAT TTC TCG CGG GTA TTT AAA AAA TGC ACC GGG GCC AGC CCG AGC GAG TTCCGT GCC GGT TGT GAA GAA AAA GTG AAT GAT GTA GCC GTC AAG TTG TCA taa-3’ |
| *cpx*P | 5’-atg CGC ATA GTT ACC GCT GCC GTC ATG GCC TCA ACG CTG GCA GTC AGT TCA TTA AGC CAC GCT GCTGAA GTC GGT TCA GGC GAT AAC TGG CAT CCG GGT GAA GAA CTT ACG CAG CGC AGT ACG CAG AGC CATATG TTC GAC GGC ATA AGT TTA ACC GAA CAT CAG CGT CAG CAG ATG CGA GAT CTT ATG CAA CAG GCCCGG CAC GAA CAG CCT CCT GTT AAT GTT AGC GAA CTG GAG ACA ATG CAT CGC CTT GTC ACC GCA GAAAAT TTT GAT GAA AAC GCT GTG CGC GCA CAG GCA GAA AAA ATG GCG AAT GAG CAA ATT GCT CGT CAGGTT GAG ATG GCA AAA GTC CGC AAC CAA ATG TAT CGC CTG TTA ACG CCG GAG CAG CAA GCG GTT TTAAAC GAG AAA CAT CAA CAA CGA ATG GAG CAG TTG CGT GAC GTG ACG CAA TGG CAA AAA AGT TCA TCGTTG AAG CTA TTG AGT AGT AGC AAC TCA CGT TCC CAGtag-3’ |
| *soh*B(TMD) | 5’-atg GAA TTG TTG TCT GAA TAT GGT TTG TTT TTG GCG AAA ATC GTT ACC GTT GTG CTA GCG ATT GCGGCG ATT GCC GCC ATT ATT GTC AAT GTT GCT CAA CGT AAT AAA CGC CAG CGT GGC GAG TTA CGG GTC AAC AAT CTC AGC-3’ |
| *yhc*B(TMD) | 5’-atg ACC TGG GAA TAT GCG CTA ATT GGG TTA GTC GTC GGC ATC ATT ATT GGT GCT GTG GCC ATG CGT-3’ |
| 17αCYP(TMD) | 5’-atg GCT CTG TTA TTA GCA GTT TTT CGT CGC CGT AAC AAT CGT CTG-3’ |
| *pel*B(SP) | 5’-atg AAA TAC CTG CTG CCG ACC GCT GCT GCT GGT CTG CTG CTC CTC GCT GCC CAG CCG GCG ATG GCC-3’ |
| *gar*P | 5’-atg ATT CTG GAC ACC GTT GAC GAA AAA AAG AAA GGC GTG CAT ACC CGC TAT TTA ATA TTA CTG ATT ATTTTT ATT GTT ACC GCC GTT AAC TAC GCC GAT CGT GCA ACG CTG TCT ATT GCT GGT ACC GAA GTG GCA AAAGAG TTG CAG TTA AGT GCG GTT TCG ATG GGT TAC ATC TTC TCC GCT TTT GGC TGG GCC TAC TTG CTG ATGCAA ATC CCC GGC GGC TGG CTG CTT GAT AAG TTT GGC TCG AAA AAA GTT TAC ACC TAC AGC CTC TTT TTCTGG TCG CTA TTC ACC TTC CTG CAA GGC TTT GTT GAT ATG TTC CCG CTG GCC TGG GCA GGG ATC TCC ATGTTC TTT ATG CGC TTT ATG CTC GGC TTC TCG GAA GCG CCA TCA TTC CCG GCG AAC GCC CGA ATT GTC GCCGCC TGG TTC CCG ACG AAA GAA CGT GGT ACT GCC TCC GCC ATC TTT AAC TCG GCG CAA TAT TTC TCG CTGGCG CTC TTT TCG CCG CTG CTT GGC TGG CTG ACT TTC GCC TGG GGC TGG GAG CAC GTC TTT ACC GTT ATGGGG GTG ATT GGT TTT GTG CTG ACG GCG CTG TGG ATC AAG TTG ATT CAT AAC CCG ACA GAT CAC CCA CGTATG TCT GCG GAA GAG CTG AAG TTT ATC TCT GAA AAT GGC GCG GTG GTC GAT ATG GAC CAC AAA AAG CCGGGC AGT GCG GCA GCA AGC GGA CCC AAA CTG CAT TAC ATC AAG CAA TTG CTC TCT AAC CGC ATG ATG CTGGGC GTA TTT TTC GGA CAA TAT TTT ATC AAC ACC ATC ACC TGG TTC TTC CTC ACC TGG TTC CCG ATT TATCTG GTG CAG GAA AAA GGC ATG TCG ATT CTG AAA GTG GGT CTG GTC GCC TCG ATT CCA GCA CTG TGT GGTTTT GCG GGC GGC GTG CTG GGA GGT GTC TTC TCG GAT TAT CTG ATC AAA CGC GGT TTA TCC CTG ACC CTGGCA CGT AAG CTA CCG ATT GTG CTG GGA ATG TTG CTG GCT TCC ACC ATC ATC TTA TGT AAC TAC ACC AACAAC ACC ACG CTG GTG GTC ATG CTG ATG GCG CTG GCT TTC TTT GGC AAA GGA TTT GGT GCG CTG GGC TGGCCG GTG ATT TCT GAC ACC GCG CCG AAA GAG ATT GTT GGC CTC TGC GGC GGC GTC TTT AAC GTC TTT GGCAAT GTT GCC TCC ATT GTC ACT CCA CTG GTG ATT GGC TAC CTG GTA AGT GAA CTG CAC TCC TTC AAT GCAGCA CTG GTT TTC GTG GGA TGT TCA GCG CTG ATG GCG ATG GTC TGC TAC CTC TTC GTA GTT GGC GAC ATTAAA CGT ATG GAA TTG CAG AAA taa-3’ |
| *yid*C | 5’-atg GAT TCG CAA CGC AAT CTT TTA GTC ATC GCT TTG CTG TTC GTG TCT TTC ATG ATC TGG CAA GCC TGGGAG CAG GAT AAA AAC CCG CAA CCT CAG GCC CAA CAG ACC ACG CAG ACA ACG ACC ACC GCA GCG GGT AGCGCC GCC GAC CAG GGC GTA CCG GCC AGT GGC CAG GGG AAA CTG ATC TCG GTT AAG ACC GAC GTG CTT GATCTG ACC ATC AAC ACC CGT GGT GGT GAT GTT GAG CAA GCT CTG CTG CCT GCT TAC CCG AAA GAG CTG AACTCT ACC CAG CCG TTC CAG CTG TTG GAA ACT TCA CCG CAG TTT ATT TAT CAG GCA CAG AGC GGT CTG ACCGGT CGT GAT GGC CCG GAT AAC CCG GCT AAC GGC CCG CGT CCG CTG TAT AAC GTT GAA AAA GAC GCT TATGTG CTG GCT GAA GGT CAA AAC GAA CTG CAG GTG CCG ATG ACG TAT ACC GAC GCG GCA GGC AAC ACG TTTACC AAA ACG TTT GTC CTG AAA CGT GGT GAT TAC GCT GTC AAC GTC AAC TAC AAC GTG CAG AAC GCT GGCGAG AAA CCG CTG GAA ATC TCC TCG TTT GGT CAG TTG AAG CAA TCC ATC ACT CTG CCA CCG CAT CTC GATACC GGA AGC AGC AAC TTC GCA CTG CAC ACC TTC CGT GGC GCG GCG TAC TCC ACG CCT GAC GAG AAG TATGAG AAA TAC AAG TTC GAT ACC ATT GCC GAT AAC GAA AAC CTG AAC ATC TCT TCG AAA GGT GGT TGG GTGGCG ATG CTG CAA CAG TAT TTC GCG ACG GCG TGG ATC CCG CAT AAC GAC GGT ACC AAC AAC TTC TAT ACCGCT AAT CTG GGT AAC GGC ATC GCC GCT ATC GGC TAT AAA TCT CAG CCG GTA CTG GTT CAG CCT GGT CAGACT GGC GCG ATG AAC AGC ACC CTG TGG GTT GGC CCG GAA ATC CAG GAC AAA ATG GCA GCT GTT GCT CCGCAC CTG GAT CTG ACC GTT GAT TAC GGT TGG TTG TGG TTC ATC TCT CAG CCG CTG TTC AAA CTG CTG AAATGG ATC CAT AGC TTT GTG GGT AAC TGG GGC TTC TCC ATT ATC ATC ATC ACC TTT ATC GTT CGT GGC ATCATG TAC CCG CTG ACC AAA GCG CAG TAC ACC TCC ATG GCG AAG ATG CGT ATG TTG CAG CCG AAG ATT CAGGCA ATG CGT GAG CGT CTG GGC GAT GAC AAA CAG CGT ATC AGC CAG GAA ATG ATG GCG CTG TAC AAA GCTGAG AAG GTT AAC CCG CTG GGC GGC TGC TTC CCG CTG CTG ATC CAG ATG CCA ATC TTC CTG GCG TTG TACTAC ATG CTG ATG GGT TCC GTT GAA CTG CGT CAG GCA CCG TTT GCA CTG TGG ATC CAC GAC CTG TCG GCACAG GAC CCG TAC TAC ATC CTG CCG ATC CTG ATG GGC GTA ACG ATG TTC TTC ATT CAG AAG ATG TCG CCGACC ACA GTG ACC GAC CCG ATG CAG CAG AAG ATC ATG ACC TTT ATG CCG GTC ATC TTC ACC GTG TTC TTCCTG TGG TTC CCG TCA GGT CTG GTG CTG TAC TAT ATC GTC AGC AAC CTG GTA ACC ATT ATT CAG CAG CAGCTG ATT TAC CGT GGT CTG GAA AAA CGT GGC CTG CAT AGC CGC GAG AAG AAA AAA TCC taa-3’ |
| **Control element** | **DNA sequence** |
| P*_cpx_*_P(+5)_ | 5-CTT TAC GTT GTT TTA CAC CCC CTG ACG CAT GTT TGC AGC CTG AAT CGT AAA CTC TCT ATC **GTTGA**-3’ |
| P*_cpx_*_P_ | 5’-CTT TAC GTT GTT TTA CAC CCC CTG ACG CAT GTT TGC AGC CTG AAT CGT AAA CTC TCT ATC-3’ |
| P*_lac_*_I_ | 5’-GAC ACC ATC GAA TGG CGC AAA ACC TTT CGC GGT ATG GCA TGA TAG CGC CC-3’ |
| P_T7_ | 5’-TAA TAC GAC TCA CTA TAG G-3’ |
| P_14_ | 5’-CTT CAT TCT ATA AGT TTC TTG ACA TCT TGG CCG GCA TAT GGT ATA ATA GGG-3’ |
| P*_pro_*_B_ | 5-GGT CTA TGA GTG GTT GCT GGA TAA CTT TAC GGG CAT GCA TAA GGC TCG TAA TAT ATA TTC-3’ |
| P*_ara_*_C_ | 5’-TGA CGC CGT GCA AAT AAT CAA TGT GGA CTT TTC TGC CGT GAT TAT AGA CAC TTT TGT TAC-3’ |
| P_BAD_ | 5’-CCA TAA GAT TAG CGG ATC CTA CCT GAC GCT TTT TAT CGC AAC TCT CTA CTG TTT CTC CAT-3’ |
| GC-linker | 5’GGT GGT TCT GGT GGT GGT TCT GGT-3’ |
| **5’UTRs** | **DNA sequence** |
| LacO operator | 5’-GGA ATT GTG AGC GGA TAA CAA TT-3’ |
| T7 5’UTR | 5’-CCC CTC TAG AAA TAA TTT TGT TTA ACT TTA AGA AGG AGA TAT ACA TA-3’ |
| LacI 5’UTR | 5’-GGA AGA GAG TCA ATT CAG GGT GGT GAA T-3’ |
| proB 5’UTR | 5’-AGG GAG ACC ACA ACG GTT TCC CTC TAC AAA TAA TTT TGT TTA ACT TTT ACT AGA GTC ACA CAG GAA AGT ACT AG-3’ |
| **Terminators** | **DNA sequence** |
| T7 terminator | 5’-CTA GCA TAA CCC CTT GGG GCC TCT AAA CGG GTC TTG AGG GGT TTT TTG-3’ |
| BioFab Terminator_FAB391 | 5’-TCG GTC AGT TTC ACC TGA TTT ACG TAA AAA CCC GCT TCG GCG GGT TTT TGC TTT TGG AGG GGC AGA AAG ATG AAT GAC TGT C-3’ |

**References**

1. Chao,Y. and Vogel,J. (2016) A 3’ UTR-Derived Small RNA provides the regulatory noncoding arm of the inner membrane stress response. *Mol. Cell*, **61**, 352–363.

2. Coussement,P., Bauwens,D., Maertens,J. and De Mey,M. (2017) Direct combinatorial pathway optimization. *ACS Synth. Biol.*, **6**, 224–232.

3. Coussement,P., Maertens,J., Beauprez,J., Van Bellegem,W. and De Mey,M. (2014) One step DNA assembly for combinatorial metabolic engineering. *Metab. Eng.*, **23**, 70–77.

4. Pédelacq,J.D., Cabantous,S., Tran,T., Terwilliger,T.C. and Waldo,G.S. (2006) Engineering and characterization of a superfolder green fluorescent protein. *Nat. Biotechnol.*, **24**, 79–88.

5. Shcherbo,D., Merzlyak,E.M., Chepurnykh,T. V., Fradkov,A.F., Ermakova,G. V., Solovieva,E.A., Lukyanov,K.A., Bogdanova,E.A., Zaraisky,A.G., Lukyanov,S., *et al.* (2007) Bright far-red fluorescent protein for whole-body imaging. *Nat. Methods*, **4**, 741–746.

6. Pritchard,M.P., Ossetian,R., Li,D.N., Henderson,C.J., Burchell,B., Wolf,C.R. and Friedberg,T. (1997) A general strategy for the expression of recombinant human cytochrome P450s in *Escherichia coli* using bacterial signal peptides: Expression of CYP3A4, CYP2A6, and CYP2E1. *Arch. Biochem. Biophys.*, **345**, 342–354.

7. Biggs,B.W., Lim,C.G., Sagliani,K., Shankar,S., Stephanopoulos,G., De Mey,M. and Ajikumar,P.K. (2016) Overcoming heterologous protein interdependency to optimize P450-mediated taxol precursor synthesis in *Escherichia coli*. *Proc. Natl. Acad. Sci. U. S. A.*, **113**, 3209–3214.
